# PIWI‐Interacting RNA HAAPIR Regulates Cardiomyocyte Death After Myocardial Infarction by Promoting NAT10‐Mediated ac^4^C Acetylation of Tfec mRNA

**DOI:** 10.1002/advs.202106058

**Published:** 2022-02-09

**Authors:** Kai Wang, Lu‐Yu Zhou, Fang Liu, Liang Lin, Jie Ju, Peng‐Chao Tian, Cui‐Yun Liu, Xin‐Min Li, Xin‐Zhe Chen, Tao Wang, Fei Wang, Shao‐Cong Wang, Jian Zhang, Yu‐Hui Zhang, Jin‐Wei Tian, Kun Wang

**Affiliations:** ^1^ Institute for Translational Medicine The Affiliated Hospital of Qingdao University College of Medicine Qingdao University Qingdao 266021 China; ^2^ Center of Diabetic Systems Medicine Guangxi Key Laboratory of Excellence and Department of Anatomy Guilin Medical University Guilin 541004 China; ^3^ State Key Laboratory of Cardiovascular Disease Heart Failure Center Fuwai Hospital National Center for Cardiovascular Diseases Chinese Academy of Medical Sciences Peking Union Medical College Beijing 100037 China; ^4^ Department of Cardiology The Second Affiliated Hospital of Harbin Medical University Harbin 150086 China

**Keywords:** ac^4^C acetylation, cardiomyocyte apoptosis, heart‐apoptosis‐associated piRNA (HAAPIR), piRNA, transcription factor EC (Tfec)

## Abstract

PIWI‐interacting RNAs (piRNAs) are abundantly expressed in heart. However, their functions and molecular mechanisms during myocardial infarction remain unknown. Here, a heart‐apoptosis‐associated piRNA (HAAPIR), which regulates cardiomyocyte apoptosis by targeting *N*‐acetyltransferase 10 (NAT10)‐mediated N4‐acetylcytidine (ac^4^C) acetylation of transcription factor EC (Tfec) mRNA transcript, is identified. HAAPIR deletion attenuates ischemia/reperfusion induced myocardial infarction and ameliorate cardiac function compared to WT mice. Mechanistically, HAAPIR directly interacts with NAT10 and enhances ac^4^C acetylation of Tfec mRNA transcript, which increases Tfec expression. TFEC can further upregulate the transcription of BCL2‐interacting killer (Bik), a pro‐apoptotic factor, which results in the accumulation of Bik and progression of cardiomyocyte apoptosis. The findings reveal that piRNA‐mediated ac^4^C acetylation mechanism is involved in the regulation of cardiomyocyte apoptosis. HAAPIR‐NAT10‐TFEC‐BIK signaling axis can be potential target for the reduction of myocardial injury caused by cardiomyocyte apoptosis in ischemia heart diseases.

## Graphical abstract

Heart‐apoptosis‐associated piRNA (HAAPIR) promotes cardiomyocyte apoptosis by regulating RNA N4‐acetylcytidine (ac4C) modifications. Mechanistically, HAAPIR promotes Tfec mRNA acetylation modifications by recruiting NAT10. This results in Tfec mRNA more stable and increases its translation efficiency, and increased expression of transcription factor TFEC, which, in turn promotes the transcription of Bik, a pro‐apoptotic factor that participates in the apoptosis process.

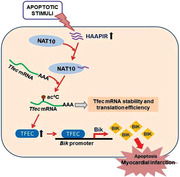

## 1 Introduction

Heart diseases are the leading cause of death around the world. Recent studies indicate that the epigenetic modifications of mRNAs play an important role in the development of cardiovascular diseases.^[^ ^1^, ^2^, ^3^ ^]^ mRNA methylation is the most abundant post‐transcriptional modification of mRNAs in mammals, and the internal methylation/demethylation of mRNA are strongly associated with both cardiac physiological and pathological processes.^[^ ^4^, ^5^, ^6^, ^7^ ^]^ N^4^‐acetylcytidine (ac*^4^* C) previously was characterized in eukaryotic tRNAs and 18S rRNA.^[^ ^8^, ^9^ ^]^ In a recent study, Arango and colleagues identify N^4^‐acetylcytidine (ac*^4^* C) as a new mRNA modification catalyzed by N‐acetyltransferase 10 (NAT10), which increases mRNA stability and enhance its translation efficiency.^[^ ^10^ ^]^ Nevertheless, how modules of ac*^4^* C modifications are dysregulated in the heart diseases remain unknown.

Piwi‐interacting RNAs (piRNAs) are a class of single‐stranded small ncRNA with 26–32 nucleotides. They are best known for their function in silencing transposons and preserve genome integrity during germ cell development, however, their functions in mammalian somatic cells remain elusive.^[^ ^11^, ^12^, ^13^, ^14^ ^]^ PiRNAs are abundantly expressed in the cardiac muscle tissue, and their expression is also highly altered during various stress conditions such as myocardial infarction and hypertrophy.^[^ ^15^, ^16^ ^]^ PiRNAs also exhibit a dynamic and a specific expression pattern during cardiac differentiation from pluripotent embryonic stem cells.^[^ ^17^ ^]^ So far, the biological functions of piRNAs in the cardiac physiology and pathogenesis of various diseases remains largely unknown. In this study, we systematically investigated previously unrecognized function of piRNA in regulating cardiomyocyte apoptosis in myocardial ischemia‐reperfusion injury and uncovered the underlying mechanism of piRNA‐mediated mRNA acetylation modification (N^4^‐acetylcytidine).

In the present study, we show that piRNA HAAPIR is a critical regulator of cardiomyocyte apoptosis in response to ischemia and reperfusion injury. HAAPIR mediates cardiomyocyte apoptosis by directly interacting with NAT10 and promoting its RNA N^4^‐acetylcytidine acetylation (ac^4^C) activity, which leads to the upregulation of TFEC expression through ac^4^C acetylation of Tfec mRNA and increase of TFEC‐dependent activation of BIK, a pro‐apoptotic factor. Our study reveals that HAAPIR‐mediated RNA ac^4^C modification has a significant contribution in the cardiomyocyte apoptosis and myocardial infarction. Thus, HAAPIR could be an efficient potential target to attenuate the myocardial infarction and ameliorate cardiac function upon ischemia and reperfusion injury.

## 2 Results

### 2.1 Identification and Characterization of Cardiac Ischaemia/Reperfusion Associated piRNA(s)

**Figure 1:**
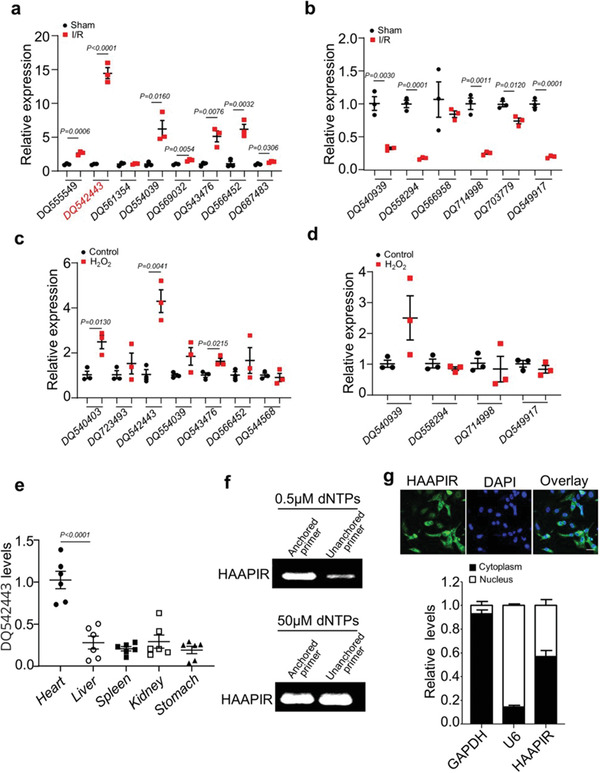
Identification of HAAPIR in cardiomyocytes. a,b) The expression levels of highly upregulated a) or downregulated b) piRNAs (selected from the Sham group in previous piRNA microarray data) in I/R injured mice hearts determined by qPCR (*n* = 3 independent experiments). c,d) qPCR analysis of highly upregulated c) or downregulated d) piRNAs in H_2_O_2_ treated cardiomyocytes selected from qPCR data in I/R injured mice hearts (*n* = 3 independent experiments). e) Relative expression level of DQ542443 in different tissues of normal adult mice as determined by qPCR (*n* = 6 independent experiments). f) Detection of 2′‐O‐methylation at the 3’ end of HAAPIR using RTL‐P approach. RT‐PCR reaction was performed with an unanchored or anchored RT primer at different concentrations of dNTPs. g) Representative images of fluorescence in situ hybridization with junction‐specific probes of HAAPIR indicates its subcellular localization (upper panel). Green represents HAAPIR and blue labels nuclei. Scale bar, 25 µm. The level of HAAPIR in the cytoplasmic or nuclear fractions of isolated cardiomyocytes as determined by qPCR. U6 and GAPDH used as internal controls (lower panel) (*n* = 3 independent experiments). Data are presented as Mean ± SEM. Two‐sided Student's *t*‐test a–d) or one‐way ANOVA test e).

To investigate the functions of piRNAs associated with apoptosis in cardiomyocyte, we selected piRNAs that are highly expressed under physiological conditions from the sham group (with raw intensity >20 000 in Sham hearts) based on our previous piRNA microarray data (GSE153445),^[^ ^3^ ^]^ and verified their expression pattern and function in ischemia/reperfusion (I/R) injured mouse hearts. We found that 19 piRNAs were increased (**Figure** **1**a; and Figure S1a,b, Supporting Information) and 5 piRNAs were decreased (Figure 1b) in I/R injured mice hearts compared to sham hearts. Further, we detected the expression levels of these piRNAs in H_2_O_2_ treated cardiomyocytes (Figure 1c,d). Among them, the expression level of DQ542443 was significantly increased in H_2_O_2_ treated cardiomyocytes. We then tested the expression levels of DQ542443 in different tissues and found that it is abundantly expressed in the heart than in other organs (Figure 1e). Comparison between cardiomyocyte and heart fibroblast, DQ542443 mainly expressed in cardiomyocyte (Figure S1c, Supporting Information). We thus speculate that DQ542443 has a potential regulatory role in I/R induced heart injury and selected DQ542443 for further study. We named this uncharacterized piRNA as heart‐apoptosis‐associated piRNA (HAAPIR) and confirmed that HAAPIR consists 2′‐*O*‐methylation at 3′‐end ^[^ ^18^ ^]^ (Figure 1f). Fluorescence in situ hybridization (FISH) test showed that HAAPIR was distributed in both the nucleus and cytoplasm of cardiomyocytes (Figure 1g).

### 2.2 Inhibition of HAAPIR Blocks H_2_O_2_‐Induced Cardiomyocyte Apoptosis

**Figure 2:**
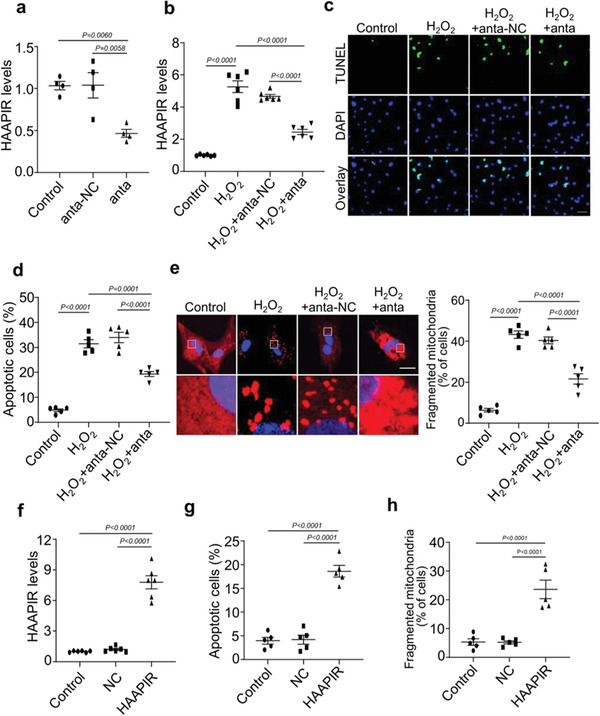
Knockdown of HAAPIR attenuates H_2_O_2_‐induced cardiomyocyte apoptosis. a) Isolated neonatal mice cardiomyocytes were transfected with HAAPIR antagomir (anta) or its negative control (anta‐NC) for 24 h. Quantitative real‐time PCR (qPCR) analysis of the expression level of HAAPIR (*n* = 4 independent experiments). b–e) Isolated neonatal mice cardiomyocytes were transfected with HAAPIR antagomir (anta) or its negative control (anta‐NC) for 24 h and then cells were treated with H_2_O_2_ for an additional 24 h. b) qPCR analysis of the expression level of HAAPIR (*n* = 6 independent experiments). c) Apoptosis was determined by the TUNEL assay. DAPI indicates Nucleus. Bar = 50 µm. d) Quantitative analysis of the percentage of apoptotic cells (*n* = 5 independent experiments). e) Cardiomyocytes were staining with MitoTracker Red (red) /DAPI (blue). Representative images show the mitochondrial fission/fusion dynamics (Left panel). Bar = 10 µm. Quantitative analysis of the percentage of cells with fragmented mitochondria (Right panel, *n* = 5 independent experiments). f–h) Isolated neonatal mice cardiomyocytes were transfected with HAAPIR agomir (HAAPIR) or its negative control (NC) for 24 h. f) qPCR analysis of the expression level of HAAPIR (*n* = 6 independent experiments). g) Quantitative analysis of the percentage of apoptotic cells (*n* = 5 independent experiments). h) Quantitative analysis of the percentage of cells with fragmented mitochondria (*n* = 5 independent experiments). Data are presented as Mean ± SEM. All data were analyzed using one‐way ANOVA.

To confirm the role of HAAPIR in the regulation of cardiomyocytes apoptosis, we used an in vitro H_2_O_2_‐induced apoptosis model. In isolated neonatal mouse cardiomyocytes, knockdown of HAAPIR (**Figure** **2**a) with inhibitor (anta) attenuated H_2_O_2_‐induced increase of HAAPIR expression (Figure 2b). HAAPIR knockdown repressed H_2_O_2_‐induced increase of cardiomyocyte apoptosis, which were detected by TUNEL (terminal deoxynucleotidyl transferase dUTP nick end labeling) assay (Figure 2c,d). The mitochondrion is a highly dynamic organelle, and mitochondrial fragmentation play an important role in mitochondrial injury during apoptosis.^[^ ^19^ ^]^ The growing body of evidence reveals that abnormal mitochondrial fusion and fission participate in the regulation of apoptosis and mitochondrial fission is involved in the initiation of apoptosis.^[^ ^20^, ^21^, ^22^ ^]^ We thus test whether HAAPIR participates in the regulation of mitochondrial fission in cardiomyocytes and found that knockdown of HAAPIR suppressed H_2_O_2_‐induced increase of mitochondrial fragmentation (Figure 2e). In contrast, the overexpression of HAAPIR (Figure 2f) caused cardiomyocyte apoptosis (Figure 2g; and Figure S1d, Supporting Information) and mitochondrial fragmentation (Figure 2h; and Figure S1e, Supporting Information).

To better simulate the condition of ischemia/reperfusion in vitro, we used the hypoxia/reoxygenation (H/R) model ^[^ ^23^, ^24^ ^]^ to induce apoptosis. In isolated neonatal mouse cardiomyocytes, the expression level of HAAPIR was increased following H/R exposure (Figure S2a, Supporting Information), and HAAPIR knockdown inhibited H/R‐induced increase of cardiomyocyte apoptosis (Figure S2b–d, Supporting Information). In addition, we also investigated the effect of HAAPIR on cardiomyocyte necrosis, and we observed that knockdown of HAAPIR could not suppress H/R‐induced necrosis in cardiomyocytes as indicated by PI‐positive cells and LDH activity (Figure S2e,f, Supporting Information). These results suggest that HAAPIR participates in the regulation of cardiomyocyte apoptosis. And it does not affect cardiomyocytes necrosis.

### 2.3 HAAPIR Deficiency Ameliorates I/R Induced Apoptosis and Myocardial Injury

**Figure 3:**
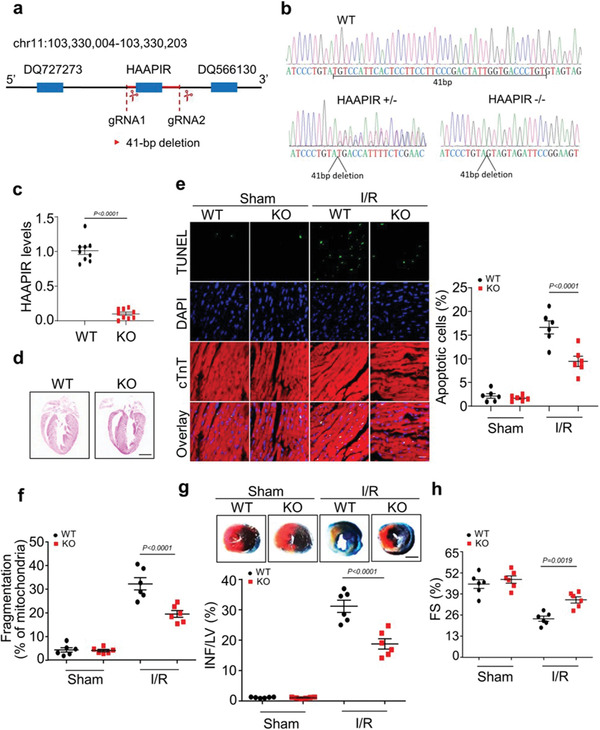
HAAPIR deficiency ameliorates ischaemia/reperfusion (I/R) induced heart injury. a) Schematic of HAAPIR location. CRISPR‐Cas9 gene editing system was used to knock out the genomic sequence of HAAPIR and generation of the mouse with mutated HAAPIR. b) HAAPIR knockout (KO) mice were genotyped by PCR and Sequencing. c) Quantitative real‐time PCR (qPCR) analysis of HAAPIR expression level in wild‐type (WT) and HAAPIR KO mice (*n* = 9 mice per group). d) Representative images of coronal sections of heart stained with hematoxylin and eosin (H&E) from WT and HAAPIR KO mice. Bar = 2 mm. e) Apoptosis was determined by the Terminal deoxynucleotidyl transferase (TdT) dUTP Nick‐End Labeling (TUNEL) assay in WT and HAAPIR KO mice heart (left panel). DAPI indicates Nucleus. Immunostaining of cTnT labels cardiomyocytes. Bar = 25 µm. Quantitative analysis of the percentage of apoptotic cardiomyocytes (right panel). *n* = 6 independent experiments. f) Quantitative analysis of the percentage of fragmented mitochondria (*n* = 6 independent experiments). g) HAAPIR KO mice exhibit reduced myocardial infarction upon I/R. WT and HAAPIR KO mice were exposed to I/R. The upper panels are representative photos of midventricular myocardial slices. The lower panel shows infarct sizes. left ventricle (LV), infarct area (INF). (*n* = 6 mice per group, Bar = 2 mm). h) Cardiac function was measured 24 h after cardiac I/R by left ventricle fractional shortening (FS) in WT and HAAPIR KO mice using echocardiography (*n* = 6 mice per group). Data are presented as Mean ± SEM. Two‐sided Student's *t*‐test c) or two‐way ANOVA test e–h).

To investigate whether HAAPIR is associated with myocardial injury and its functional role in vivo, we generated HAAPIR knockout (HAAPIR KO) mice using CRISPR/Cas9 technology (**Figure** **3**a). Genotyping by sequencing and gene expression analysis by RT‐PCR confirmed the loss of HAAPIR in KO mice (Figure 3b,c). We did not observe any morphological or functional phenotype changes in HAAPIR KO hearts under basal conditions (Figure 3d). Compared with wild‐type (WT) hearts, HAAPIR KO mice hearts exhibited a dramatic reduction of cardiomyocyte apoptosis and fragmented mitochondria with I/R injury (Figure 3e,f). Furthermore, a reduced infarct size (Figure 3g) and an improved ventricle function (Figure 3h) were observed in HAAPIR KO hearts compared to WT hearts with I/R injury. Together, these results indicate that HAAPIR deficiency blocks cardiomyocytes apoptosis and attenuates myocardial dysfunction caused by I/R injury.

### 2.4 HAAPIR Binds to NAT10 and Regulates Its N^4^‐Acetylcytidine Acetylation Activity

**Figure 4:**
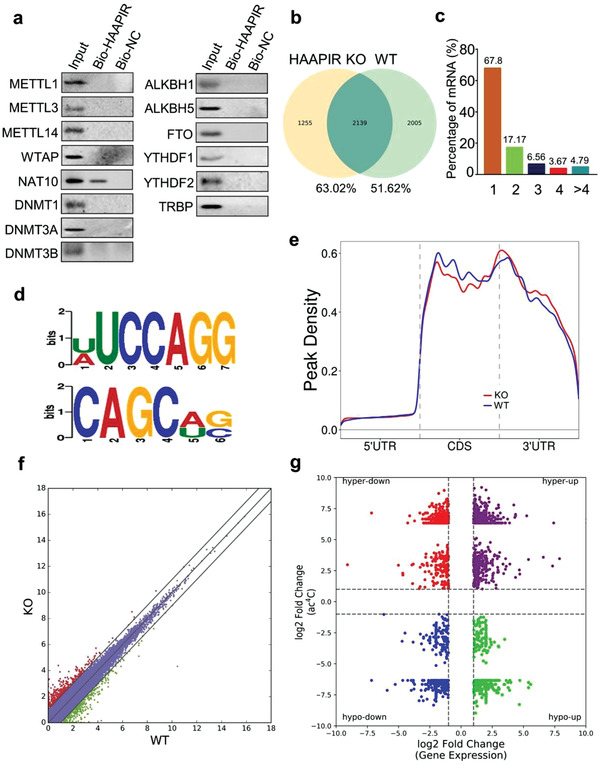
HAAPIR binds to NAT10 and influences its N^4^‐acetylcytidine acetylation function. a) Cardiomyocytes were harvested and RNA pull‐down assay was performed using Bio‐ HAAPIR or Bio‐NC. Associated proteins were pulled down with streptavidin beads and bound levels of METTL1, METTL3, METTL14, NAT10, WTAP, DNMT1, DNMT3A, DNMT3B, ALKBH1, ALKBH5, FTO, YTHDF1, YTHDF2, and TRBP were analyzed by western blot. Representative image from three independent experiments. b–g) Ac^4^C acetylated RNA immunoprecipitation and sequencing (acRIP‐seq) was performed in HAAPIR knockout (KO) and wild‐type (WT) mice hearts. b) Numbers of ac^4^C peaks detected in HAAPIR KO (left circle) and WT (right circle) mice hearts. c) Percentage of mRNAs with different numbers of ac^4^C peaks. d) Sequence motifs enriched within ac^4^C peaks identified by acRIP‐seq. e) Metagene profile showing the distribution of ac^4^C peaks across the length of transcripts composed of three rescaled nonoverlapping segments 5′UTR, CDS, and 3′UTR in HAAPIR KO and WT mice hearts. f) Scatter plot of differential expression of mRNAs assessed from RNA‐seq data. Red dots denote up‐regulated genes and green dots denote down‐regulated genes. g) Correlation between the level of gene expression (overall transcript) and changes in ac^4^C level in HAAPIR KO mice hearts compared to WT.

We next sought to investigate the molecular mechanisms by which HAAPIR regulates cardiomyocyte apoptosis. Recently, epigenetic modifications of mRNAs have a great impact on multiple fundamental biological processes.^[^ ^25^, ^26^, ^27^ ^]^ We previously demonstrated that piRNA is involved in the regulation of METTL3‐dependent m^6^A methylation.^[3^^]^ To further investigate the functional relevance of piRNA and mRNA epigenetic regulation, we examined the epigenetics‐related molecules interacting with HAAPIR in cardiomyocytes using biotinylated HAAPIR and performed RNA pull‐down assay. Among those molecules, only N‐acetyltransferase 10 (NAT10), an RNA cytidine acetyltransferase, was detected in biotinylated HAAPIR pulldown product (**Figure** **4**a), which indicates that HAAPIR bound with NAT10 in cardiomyocytes. Reversely, RIP followed by qPCR showed the enrichment of HAAPIR in NAT10, but not in other protein‐RNA precipitates (Figure S3a,b, Supporting Information), which validates the direct interaction HAAPIR with NAT10 in vivo. Also, the overexpression or knockout of HAAPIR did not affect the expression levels of mRNA and protein of NAT10 in cardiomyocytes (Figure S3c–f, Supporting Information). Together, these results reveal that HAAPIR interacts with NAT10 and might be associated with the regulation of N^4^‐acetylcytidine acetylation activity.

### 2.5 ac^4^C Acetylation in HAAPIR Knockout Mice Hearts

To elucidate the molecular mechanism by which HAAPIR regulates N^4^‐acetylcytidine modification, we performed acetylated RNA immunoprecipitation and sequencing (acRIP‐seq) in HAAPIR KO and WT mice hearts (Table S1, Supporting Information). HAAPIR KO and WT mice hearts shared 2139 common ac^4^C peaks, which accounted for 63.02% of total acetylated peaks in HAAPIR KO hearts and 51.62% in WT hearts (Figure 4b). Ac^4^C mostly occurred in mRNAs and majority of mRNAs contain one or two ac^4^C peaks (Figure 4c). The sequential analysis of ac^4^C peaks showed that U(A)UCCAGG and CAGCA(U)G(C) motifs were highly enriched within ac^4^C sites (Figure 4d). Ac^4^C peaks were predominantly distributed in coding sequences (CDSs), near stop codon and 3’ untranslated regions (3’UTRs) (Figure 4e; and Figure S4a, Supporting Information). Gene Ontology (GO) analysis showed that genes with upregulated ac^4^C modification were mainly involved in cardiovascular system development, positive regulation of signal transduction and cell death. (Figure S4b, Supporting Information). Genes with downregulated ac^4^C modification were mainly involved of regulation of molecular function, regulation of cell differentiation, and cell death (Figure S4c, Supporting Information). These results indicate that the genes with ac^4^C modification are related to cellular function or regulation of gene expression in heart. To determine the relationship between ac^4^C modification and gene expression, the RNA‐seq data was performed in HAAPIR KO and WT mice hearts (Figure 4f; and Table S2, Supporting Information). For correlating the gene expression level with ac^4^C modification level, we plotted ac^4^C peaks data against RNA‐seq data of gene expression (Figure 4g).

### 2.6 HAAPIR Promotes NAT10‐Mediated ac^4^C Modification and Expression of Tfec mRNA

**Figure 5:**
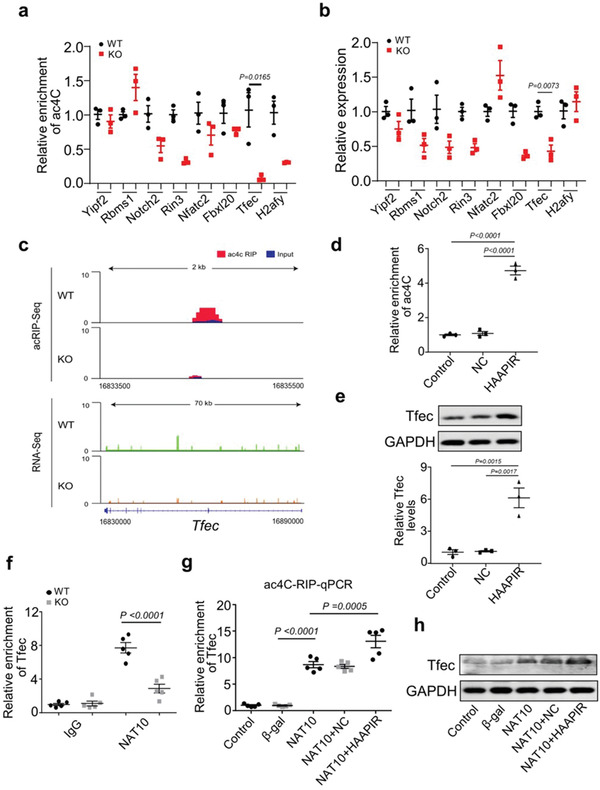
HAAPIR promotes ac^4^C modification and expression of Tfec by targeting NAT10. a) Ac^4^C acetylated RNA immunoprecipitation and Quantitative real‐time PCR (acRIP‐qPCR) validation of ac^4^C modification levels of genes which selected from the results of acRIP‐seq and mRNA‐seq data in HAAPIR knockout (KO) and wild‐type (WT) mice hearts (*n* = 3 mice per group). b) qPCR validation of the expression levels of genes which selected from the results of acRIP‐seq and mRNA‐seq data in HAAPIR KO and WT mice hearts (*n* = 3 mice per group). c) Integrative Genomics Viewer (IGV) tracks displaying results of ac^4^C‐seq (upper panels) and RNA‐seq (lower panels) read distribution in Tfec mRNA of HAAPIR KO and WT mice hearts. d) acRIP‐qPCR analysis in isolated cardiomyocytes treated with or without HAAPIR agomir or agomir‐NC (NC) shows the ac^4^C modification level in Tfec mRNA. e) Expression levels of Tfec protein (upper panel) and mRNA (lower panel) in cardiomyocytes treated with HAAPIR agomir or NC (*n* = 3 independent experiments). f) RIP‐qPCR analysis in WT or HAAPIR KO mice hearts shows the level of Tfec mRNA binding to NAT10 (*n* = 5 mice per group). g) Ac^4^C enrichment level in Tfec mRNA was detected in cardiomyocytes infected with adenovirus harboring NAT10 or *β*‐gal and transfected with HAAPIR agomir or NC (*n* = 5 independent experiments). h) Western blot assay shows the protein level of TFEC in cardiomyocytes infected with adenovirus harboring NAT10 or *β*‐gal and transfected with HAAPIR agomir or NC. Data are presented as Mean ± SEM. Two‐sided Student's *t*‐test a,b), one‐way ANOVA test d,e,g) or two‐way ANOVA test f).

Next, we conducted acRIP‐qPCR and qPCR assays for some differentially acetylated genes and differentially expressed genes reported to be associated with apoptosis (**Figure** **5**a,b). Among them, TFEC had the lowest ac^4^C modification and dramatically decreased expression in HAAPIR KO hearts (Figure 5a–c; and Figure S4d, Supporting Information). In contrast, ac^4^C enrichment at Tfec was increased (Figure 5d) along with an increase in the levels of Tfec mRNA and protein (Figure 5e) in HAAPIR overexpressed cardiomyocytes. Based on the above results, we then chose Tfec as a candidate ac^4^C target in the regulation of cardiomyocyte apoptosis for further studies.

We then investigated how HAAPIR upregulates Tfec expression. In HAAPIR KO hearts, the binding of NAT10 to Tfec mRNA was remarkably decreased compared to WT mice hearts (Figure 5f). In addition, the enforced expression of NAT10 in cardiomyocytes increased ac^4^C modification in Tfec mRNA and elevated Tfec protein level, and these effects were reinforced upon HAAPIR overexpression (Figure 5g,h). These results indicate that HAAPIR mediates ac^4^C modification in Tfec mRNA through NAT10, and this acetylation modification enforce its stability and translation capacity.

### 2.7 Inhibition of Tfec Attenuates Cardiomyocyte Apoptosis In Vitro and In Vivo

**Figure 6:**
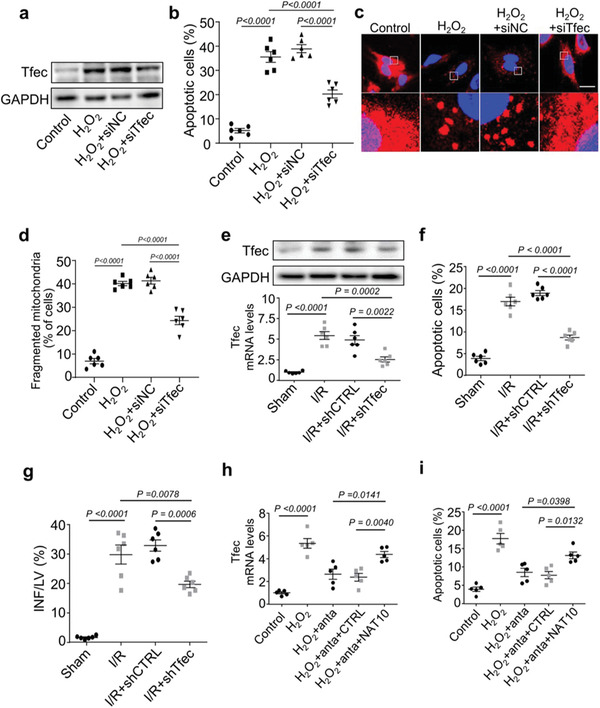
Inhibition of Tfec attenuates cardiomyocyte apoptosis in vitro and in vivo. a–d) Isolated neonatal mice cardiomyocytes were transfected with Tfec siRNA (si‐Tfec) or its negative control (si‐NC) for 24 h and then cells were treated with H_2_O_2_ for an additional 24 h. a) Representative western blot showing the expression of TFEC. b) The percentage of apoptotic cardiomyocytes was determined by TUNEL assay (*n* = 6 independent experiments). c) Cardiomyocytes were stained with MitoTracker Red (red) /DAPI (blue). Representative images show the mitochondrial fission/fusion dynamics. Bar = 10 µm. d) Quantitative analysis of the percentage of cells with fragmented mitochondria (*n* = 6 independent experiments). e–g) AAV9‐Tfec‐shRNA (shTfec) or AAV9‐control (shCTRL) were injected into mice and I/R induced heart injury was performed 3 weeks after the injection. e) The protein (upper panel) and mRNA (lower panel) levels of Tfec were detected by western blot assay and quantitative real‐time PCR (qPCR) assay (*n* = 6 mice per group). f) The percentage of apoptotic cardiomyocytes was determined by TUNEL assay (*n* = 6 mice per group). g) The infarct sizes after I/R induced heart injury were indicated by the ratio of infarct area (INF)/left ventricle (LV) (*n* = 6 mice per group). h,i) Cardiomyocytes were infected with adenovirus harboring NAT10 (NAT10) or its control (CTRL) and transfected with HAAPIR antagomir (anta) for 24 h, and then cells were treated with H_2_O_2_ for an additional 24 h. h) The mRNA levels of Tfec were detected by qPCR (*n* = 5 independent experiments). i) The percentage of apoptotic cardiomyocytes was determined by TUNEL assay (*n* = 5 independent experiments). Data are presented as Mean ± SEM. All data were analyzed using one‐way ANOVA.

TFEC is a member of the MiTF/TFE (Microphthalmia/TFE) family of basic/helix‐loop‐helix/leucine zipper (bHLH‐LZ) transcription factors.^[^ ^28^, ^29^ ^]^ The MiTF/TFE are known to play an important role in organelle biogenesis and energy metabolism,^[^ ^29^ ^]^ while the function of TFEC remains unknown in cardiac tissue. In cardiomyocytes, Tfec expression were increased in response to H_2_O_2_ stimulus (**Figure** **6**a), and silencing of Tfec blocked H_2_O_2_‐induced cardiomyocyte apoptosis (Figure 6b; and Figure S5a, Supporting Information) and mitochondrial fragmentation (Figure 6c,d). In vivo, the levels of Tfec mRNA and protein were increased following myocardial I/R injury (Figure 6e) and Tfec knockdown markedly reduced Tfec levels (Figure 6e), cardiomyocyte apoptosis (Figure 6f; and Figure S5b, Supporting Information)) and infarct size (Figure 6g). In addition, knockdown of Tfec suppressed H/R‐induced apoptosis (Figure S6a,b, Supporting Information) and did not affect necrosis (Figure S6c,d, Supporting Information) in cardiomyocytes. Further, HAAPIR knockdown attenuated H_2_O_2_‐induced increase of Tfec expression (Figure 6h; and Figure S7a,b, Supporting Information) and apoptosis (Figure 6i), while these effects were attenuated upon NAT10 overexpression (Figure 6h,i). The enforced expression of HAAPIR increased Tfec protein levels and apoptosis in cardiomyocytes, and this increase was reversed upon NAT10 knockdown (Figure S7c,d, Supporting Information). Together, our data suggest that Tfec participates in the regulation of cardiomyocyte apoptosis, and Tfec is direct downstream molecule of HAAPIR and NAT10.

### 2.8 Tfec Regulates Bik Expression During Cardiomyocyte Apoptosis

**Figure 7:**
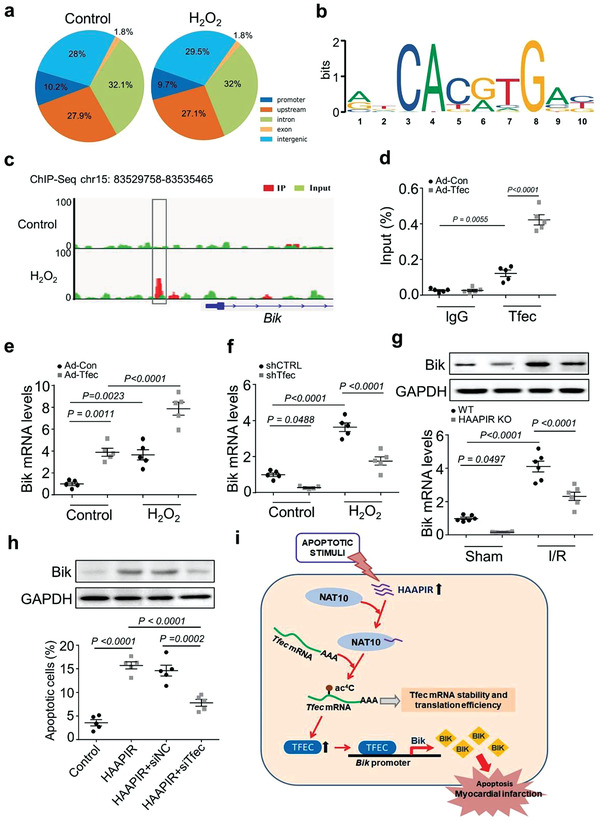
Tfec regulates Bik expression during cardiomyocyte apoptosis. a–c) ChIP‐seq analysis was performed using flag‐tagged Tfec in cardiomyocytes treated with or without H_2_O_2_. a) Pie chart depicting the genomic distribution of Tfec‐enrichment. b) Motif analysis of the Tfec bound regions. c) Genome browser view showing Tfec ChIP‐seq signal around Bik. A Tfec peak in the promoter region on Bik was indicated. d) Isolated neonatal cardiomyocytes were infected with adenovirus harboring Tfec (Ad‐Tfec) or its negative control (Ad‐Con) for 24 h. CHIP‐qPCR assay was performed using antibodies against Tfec or IgG (*n* = 5 independent experiments). e,f) Cardiomyocytes were infected with Ad‐con or Ad‐Tfec e), control‐siRNA (sh‐CTRL) or Tfec‐siRNA (sh‐Tfec) f), and treated with H_2_O_2_ for 24 h. Bik mRNA levels were evaluated by qRT‐PCR (*n* = 5 independent experiments). g) HAAPIR knockout (KO) and wild‐type (WT) mice were exposed to I/R injury. The protein (upper panel) and mRNA (lower panel) levels of Bik were detected by western blot assay and qPCR, respectively (*n* = 6 mice per group). h) Cardiomyocytes were transfected with Tfec siRNA (siTfec) or its negative control (siNC) and transfected with HAAPIR agomir (HAAPIR) for 24 h. The protein levels of Bik were detected by western blot assay (upper panel). Quantitative analysis of the percentage of apoptotic cells were determined by TUNEL assay (*n* = 5 independent experiments). i) Model of HAAPIR function in apoptotic signaling. HAAPIR participates in the regulation of cardiac apoptosis through targeting NAT10/Tfec/Bik pathway. In our model, HAAPIR promotes NAT10‐mediated ac^4^C acetylation of Tfec mRNA transcript, which leads to an increase of Tfec expression, promotes Tfec‐induced Bik expression and thus leads to cardiomyocytes apoptosis. Data are presented as Mean ± SEM. All data were analyzed using one‐way ANOVA.

To explore the downstream signaling of Tfec, we performed a Chromatin Immunoprecipitation Sequencing (ChIP‐seq) assay using TFEC antibody in Tfec‐overexpressed cardiomyocytes subjected to H_2_O_2_ stimulation. ChIP‐seq identified thousands of differentially TFEC‐bound chromatin regions (**Figure** **7**a). Motif discovery using these TFEC‐bound regions yielded the consensus TFEC motif (CACGTG), providing validation for the ChIP‐seq dataset (Figure 7b). To further identify TFEC‐mediated downstream genes, we performed a transcriptome analysis (RNA‐Seq), and RNA‐Seq analysis identified 694 upregulated and 959 downregulated differentially expressed mRNAs in H_2_O_2_ treated Tfec‐overexpressed cardiomyocytes compared to control (Figure S8a, Supporting Information). The intersection of genes associated with TFEC‐bound chromatin regions and regulated downstream of TFEC in differential expression datasets contained 376 genes (Figure S8b and Table S3, Supporting Information). In order to narrow the number of TFEC‐mediated genes, we screened genes associated with TFEC‐bound promoter region and genes with apoptosis regulation function. We focused on Bik, a pro‐apoptotic protein and a member of the BCL2 family.^[^ ^30^, ^31^, ^32^ ^]^ ChIP‐seq analysis revealed a significant TFEC‐binding promoter region in the Bik gene (Figure 7c). We validated TFEC binding to the identified sequence by ChIP‐qPCR and confirmed marked enrichment of Bik promotor region over normal IgG following TFEC overexpression (Figure 7d). Overexpression of Tfec dramatically increased H_2_O_2_‐induced Bik mRNA expression (Figure 7e), whereas knockdown of TFEC reduced the increase of H_2_O_2_‐stimulated Bik expression (Figure 7f), suggesting this transcription factor TFEC stimulates Bik expression. Consistent with these in vitro findings, Bik mRNA and protein expression were remarkably decreased in HAAPIR KO mice hearts with I/R injury compared to WT hearts (Figure 7g). In addition, Knockdown of Bik attenuated H_2_O_2_‐induced cardiomyocyte apoptosis (Figure S8c, Supporting Information) and mitochondrial fragmentation (Figure S8d, Supporting Information). In cardiomyocytes, HAAPIR increased the levels of Bik protein and apoptosis, while these increases were reversed upon TFEC knockdown (Figure 7h) or NAT10 knockdown (Figure S8e, Supporting Information).

To further validate the effect of activating the HAAPIR‐NAT10‐TFEC‐BIK pathway on cardiac ischaemia/reperfusion injury, we examined the effect of TFEC knockdown on I/R‐induced myocardial injury in HAAPIR KO mice. The results showed that HAAPIR knockout inhibited I/R‐induced increases of Bik expression, cardiomyocytes apoptosis and infarct size. And knockdown of TFEC enhanced the effects of HAAPIR knockout in I/R‐treated mice hearts (Figure S9a–c, Supporting Information). Similarly, echocardiography analysis revealed that the left ventricular function was further ameliorated in TFEC knockdown‐treated HAAPIR KO mice with I/R (Figure S9d, Supporting Information). Together, these results suggest that TFEC promotes Bik expression through its direct binding to the promoter region of Bik, and TFEC and Bik are direct downstream molecules of HAAPIR in the regulation of cardiomyocyte apoptosis and myocardial infarction with I/R injury.

## 3 Discussion

Here, we have identified that HAAPIR, an apoptosis‐associated piRNA, promotes cardiomyocyte apoptosis by regulating RNA N^4^‐acetylcytidine (ac^4^C) modifications. Mechanistically, HAAPIR promotes Tfec mRNA acetylation modifications by recruiting NAT10. This results in Tfec mRNA more stable and increases its translation efficiency, and increased expression of transcription factor TFEC, which, in turn promotes the transcription of Bik, a pro‐apoptotic factor that participates in the apoptosis process (Figure 7i). Overall, the findings in our study propose that HAAPIR is an important pro‐apoptotic factor by RNA epigenetic modification of Tfec mRNA.

Previous reports demonstrate that many piRNAs are abundantly and differentially expressed in cardiac tissue during myocardial infarction and the progression of hypertrophy.^[^ ^15^, ^16^ ^]^ The dynamic expression pattern of piRNA was observed during cardiac differentiation from pluripotent embryonic stem cells.^[^ ^17^ ^]^ The somatic cell functions of most piRNAs have not been well characterized to date, and hence their functions in the cardiac physiological and pathological process are seldom identified. Here, our results show that HAAPIR expression in the heart tissue and cardiomyocytes is relatively high compared to other organs and cardiac fibroblasts, respectively, indicating that HAAPIR has cardiac‐specific functions. Here, we found that HAAPIR directly binds to NAT10, an RNA acetyltransferase, but not affects its expression or stability. Generally, piRNAs regulated the gene expression rely on PIWI proteins.^[^ ^13^, ^33^ ^]^ Hence, we speculate that HAAPIR, PIWI proteins, and NAT10 form complex, and influences its RNA acetylation functions. Notably, our findings demonstrate that NAT10‐dependent ac^4^C mRNA acetylation is essential for controlling cardiomyocyte apoptotic response. However, HAAPIR acts as a pro‐apoptotic factor, and influences RNA acetylation functions of NAT10 during cardiomyocyte apoptosis.

In present study, we show that Tfec mRNA is a downstream ac^4^C acetylation target of HAAPIR‐NAT10 complex, which upregulates its expression. We observed that overexpression of HAAPIR increase ac^4^C acetylation modification of Tfec mRNA along with the increase of its mRNA and protein level. Hence, we speculate ac^4^C acetylation modification of Tfec mRNA increase Tfec expression through modulation of its stability and translation, which is consistent with the results from previous reports.^[^ ^10^, ^34^ ^]^ TFEC is a MiTF/TFE (Microphthalmia/TFE) family transcription factors.^[^ ^28^, ^29^ ^]^ The MiTF/TFE family member play an important role in organelle biogenesis and energy metabolism,^[^ ^29^ ^]^ and regulates cardiac growth and hypertrophy.^[^ ^35^ ^]^ The expression level of TFEC is low in heart compared to other organs,^[^ ^36^ ^]^ and antioxidant treatment significantly suppressed the expression of Tfec in the infarcted myocardium.^[^ ^37^ ^]^ Our results show that Tfec knockdown reduces pathological stimuli‐induced cardiomyocyte apoptosis, which imply that TFEC may serve as a pro‐apoptotic transcription factor in the development of heart diseases.

Our present study demonstrates that TFEC can positively regulate the transcription of Bik during H_2_O_2_‐induced cardiomyocyte apoptosis and I/R‐induced myocardial injury. BIK is a member of the BH3‐only pro‐apoptotic proteins,^[^ ^38^ ^]^ which is predominantly localized in the ER^[^ ^39^ ^]^ and induces apoptosis through the mitochondrial pathway.^[^ ^40^ ^]^ BIK can mobilize calcium influx from ER to the mitochondria, and induce apoptosis and the remodeling of the mitochondrial cristae.^[^ ^40^, ^41^ ^]^ BIK is also reported that involves in Osteopontin‐stimulated cardiomyocyte apoptosis by mitochondrial death pathway.^[^ ^42^ ^]^ Consist with these reports, our present data shows that Bik participates in the regulation of cardiomyocyte apoptosis. Besides, we demonstrated that Bik acts as a transcriptional target of TFEC, participating in the regulation of myocardial injury.

Thus, our study reinforces the crucial role of mRNA ac^4^C modifications in regulation of cardiomyocyte apoptosis and provides new insights into the mechanisms of piRNA directed post‐transcriptional gene regulation. Our findings reveal that, except mRNA methylation,^[^ ^3^ ^]^ mRNA acetylation modifications also involve in the pathological process of heart diseases. Further, a remarkable improvement of cardiac function along with a significant reduction of infarction area in HAAPIR‐deficient mice hearts suggests that targeting HAAPIR could be a useful therapeutic strategy to alleviate ischemia injury in the heart.

## Conflict of Interest

The authors declare no conflict of interest.

## Author Contributions

K.W., L.‐Y.Z., F.L., L.L., and J.J., contributed equally to this work. K.W., J.T., and Y.Z. designed research. K.W., L.Z., F.L., L.L., J.J., P. T., C.L., X.L., X.C., T.W., F.W., and S.W. performed experiments. K.W., L.Z., F.L., L.L., J.T., and J.Z. analyzed the data. F.L., L.Z., K.W., and J. Z. wrote the manuscript.

## Supporting information

Supporting Information

Supplemental Table 1

Supplemental Table 2

Supplemental Table 3

Supplemental Table 4

## Data Availability

The data that support the findings of this study are available in the supplementary material of this article.
