# Supplementary material for: PIWI‐Interacting RNA HAAPIR Regulates Cardiomyocyte Death After Myocardial Infarction by Promoting NAT10‐Mediated ac4C Acetylation of Tfec mRNA
Source: Adv Sci (Weinh). 2022 Feb 9;9(8):2106058. doi: 10.1002/advs.202106058 (PMC8922123; doi:10.1002/advs.202106058)
Supplement: Supplementary file 1 — Supporting Information [file ADVS-9-2106058-s001.pdf]

ADVANCED SCIENCE
Open Access

Supporting Information

for *Adv. Sci.*, DOI 10.1002/advs.202106058

PIWI-Interacting RNA HAAPIR Regulates Cardiomyocyte Death After Myocardial Infarction by Promoting NAT10-Mediated ac^{4}C Acetylation of Tfec mRNA

Kai Wang, Lu-Yu Zhou, Fang Liu, Liang Lin, Jie Ju, Peng-Chao Tian, Cui-Yun Liu, Xin-Min Li, Xin-Zhe Chen, Tao Wang, Fei Wang, Shao-Cong Wang, Jian Zhang, Yu-Hui Zhang, Jin-Wei Tian* and Kun Wang**

ADVANCED SCIENCE
Open Access

Supporting Information

for *Adv. Sci.,* DOI: 10.1002/advs.202106058

PIWI-Interacting RNA HAAPIR Regulates Cardiomyocyte Death After Myocardial Infarction by Promoting NAT10-Mediated ac^4C Acetylation of Tfec mRNA

Kai Wang, Lu-Yu Zhou, Fang Liu, Liang Lin, Jie Ju, Peng-Chao Tian, Cui-Yun Liu, Xin-Min Li, Xin-Zhe Chen, Tao Wang, Fei Wang, Shao-Cong Wang, Jian Zhang, Yu-Hui Zhang, Jin-Wei Tian,* and Kun Wang**

SUPPLEMENTARY MATERIAL

Primary cardiomyocytes culture and treatment. The neonatal mouse cardiomyocytes (NMCs) were isolated from 1 to 2 days old mice, after dissection hearts were washed clean and minced in PBS buffer. Tissues were then dispersed in a series of incubations at 37°C in a digestive solution containing pancreatin (Sigma, St. Louis, Missouri, USA) and collagenase II (Worthington, Lakewood, USA). Subsequent supernatants were collected and centrifuged at 200×g for 5 min. After centrifugation, cells were resuspended in Dulbecco's modified Eagle's medium/F-12 (DMEM/F12) (Gibco, CA, USA) containing 5% heat-inactivated Fetal bovine serum (ExCell, Shanghai, China), 1% Penicillin-Streptomycin Liquid (Solarbio, Beijing, China). After 1.5 h pre-plated at 37°C differential adherent isolation and purification of NMCs. Add bromodeoxyuridine (Brdu) (Sigma, St. Louis, Missouri, USA) in the cell suspension to inhibit the growth of non-cardiac myocytes. After 48h, change the medium and select the cells which in good condition for the subsequent experiments. Cells were treated with 150 μM H_2O_2 (Sigma, St. Louis, Missouri, USA) for the indicated periods of time. Hypoxia/reoxygenation (H/R) treatment of NMCs requires replacing sugar-free DMEM/F12 (Meilunbio, MA0598, Dalian, China) medium 12 h in advance, and then the cells are cultured in a low oxygen environment at 37°C for 6 h. After the hypoxia treatment, it was replaced with DMEM/F12 medium containing 5% serum, reoxygenated in an incubator with 5% CO2 and 95% O2 at 37°C for 6 h.

Animal experiments and Generation of HAAPIR knockout (HAAPIR KO) mice. Adult male mice of 8-10 weeks were used in this study. Mice were raised at 24±2℃ with an interval of 12 hours for a dark or light cycle. All animal experiments were conducted according to the protocols approved by Laboratory Animal Welfare Ethics Committee of Qingdao University, Qingdao, China.

HAAPIR knockout mice were generated by using clustered regularly interspaced short palindromic repeats associated protein 9 (CRISPR/Cas9) gene-editing system (Cyagen Biosciences Inc. Guangzhou, China). HAAPIR +/- mice were interbred to generate KO mice (HAAPIR -/-), which were used for further studies. Mice were genotyped by PCR with forward primer: GACCAACCCCAACAGTCTCTCATC and reverse primer: TCCGTGGCTGTCTTATCATGGATC. The PCR products were further sequenced to verify that the HAAPIR was correctly deleted. All experiments were performed on HAAPIR-/- mice and their WT littermates.

I/R injury. For the establishment of I/R injury, the mice were anesthetized by intraperitoneal injection of 4% chloral hydrate (10ml kg-1 mouse). Under aseptic conditions, the heart was exposed by left thoracotomy, and the left anterior descending (LAD) coronary artery was ligated in the temporal region for 45 min of ischemia and followed by 3 h reperfusion as previously described[1]. In the Sham-operated group, a similar procedure except the snare was left untied. For Tfec knockdown in vivo, the Tfec knockdown vector was constructed by recombinant serum type 9 adeno associated virus system (AAV9) (Hanbio, Shanghai, China). Mice

were given tail vein injection 3 weeks before Sham or I/R operation (5×10^{10} vg/mouse). The hearts were collected in the in-vivo study after 45mins ischemia and 3 hours reperfusion.

Echocardiography measurement and Myocardial infarct size measurement. After I/R or Sham operation, echocardiographic measurements were performed using the Vevo2100 imaging system (Visual Sonic) to assess cardiac function. After echocardiography, the mice were sacrificed and the hearts were removed for further study. After echocardiography, 1% Evans blue dye (Sigma, St. Louis, Missouri, USA) was injected into jugular vein of the mice, and the hearts were removed and transversely sectioned serially into 2 mm-thick slices. Then, the heart slices were incubated in 1.5% 2,3,5-triphenyltetrazolium chloride (Sigma, St. Louis, Missouri, USA) at 37℃ for 10-30 min under dark conditions. After removal, PBS was washed for 5 min. 4% paraformaldehyde (Servicebio, G1101, Wuhan, China) was fixed at room temperature for 15-25min, and the infarcted area was taken by camera (Nikon-D5600) and analyzed by ImageJ 7.0 (NIH, Boston, USA).

Transfection of HAAPIR agomir and antagomir. All HAAPIR agomir or antagomir and their negative controls were obtained from Gene Pharma (Shanghai, China). Agomir or antagomir of HAAPIR was transfected into cardiomyocytes using Lipofectamine 3000 (Invitrogen, NY, USA). Taking 6-well plate as an example. Specifically, mix 5μl of Lipofectamine 3000 and an equal volume of solution

containing agomir or antagomir of HAAPIR with 125 μl of serum-free DMEM/F12, respectively, and let stand for 5 min, then mix the two. Let stand for 15-20 min and add to a well in a 6-well plate containing 2ml DMEM/F12 medium. The HAAPIR agomir sequence is 5'-UCCAUUCACUCCUUCCUUCCCGACUAUUGG-3', the agomir negative control (agomir-NC) sequence is 5'-UUCUCCGAACGUGUCACGUTT-3'. The antagomir sequence is 5'-CCAAUAGUCGGGAAGGAAGGAGUGAAUGGA-3', the antagomir negative control (anta-NC) sequence is 5'-CAGUAUUUUGUGUAGUACAA-3'.

RNA interference (RNAi). Small interfering RNA (siRNA) oligonucleotides specific for Tfec, NAT10 and Bik were purchased from Gene Pharma Co. Ltd (Shanghai, China). The siRNA sequences used were: Tfec siRNA, 5'-AUUAGCUGAAGAAAUGCCCTT-3', Tfec-siRNA negative control (si-NC), 5'-ACGUGACACGUUCGGAGAATT-3'; Bik siRNA, 5'-UGAAGCUGCAAAUACCAGGTT-3', Bik-siRNA negative control (si-NC), 5'-ACGUGACACGUUCGGAGAATT-3'. Transfection of siRNAs was performed using Lipofectamine TM 2000 (Invitrogen) according to the manufacturer's instructions.

Apoptosis assays and Mitochondrial staining. Apoptosis was determined by the TdT mediated dUTP Nick End Labelling (TUNEL) using a kit from Yeasen (Shanghai, China). The operation procedures were in accordance with the instructions

of kit. In brief, the fixed tissue sections or cell climbing slices were treated with Proteinase K solution or 0.2% Triton X-100 solution in PBS for 5-10min. Rinse with PBS 3 times, 5 min each. Add 100μl 1xEquilibration Buffer (deionized water configuration) to each well, incubate at room temperature for 10-30 min. After absorbing 1xEquilibration Buffer, add 50μl TdT incubation buffer, cover with plastic cover slides, put in a wet box under dark conditions, incubate at 37℃ for 1 h; remove cover slides, add PBS to wash 3 times, 5 min each; add 100μl PBS containing 5% BSA and 0.1% Triton X-100, rinse again 3 times, 5 min each, to reduce the background. Add fluorescence quenching seal containing 4, 6-diamidino-2-phenylindole (DAPI) (Solarbio, Beijing, China), observe under fluorescence microscope (Nikon, ECLIPSE Ti-S).

The mitochondrial staining as previously described[2]. Cells were plated onto the cover slips coated with 0.01% poly-L-lysine. After treatment with transfection or H_2O_2, cells were incubated in 0.02 μM Mito Tracker Red CMXRos (Yeasen, Shanghai, China) at 37℃ for 30 min under dark conditions. After PBS washing three times, 4% paraformaldehyde was fixed at room temperature for 15-25min. PBS was washed three times, and the cells were counterstained with DAPI. Mitochondria were analyzed under a confocal fluorescence microscope (Leica, TCS SP8).

Fluorescence in situ hybridization (FISH). The distribution of HAAPIR in cardiomyocytes was detected by the FISH kit (Gene Pharma, Shanghai, China). After 4% paraformaldehyde was fixed, 0.1% Triton X-100 was permeated at 25℃ for

15min. PBS was washed three times, 5 min each time. After dehydration and drying with gradient ethanol according to the instructions, the hybrid buffer was used to incubate at 37℃ for 2 h under dark conditions. Follow the kit instructions and incubate overnight in probe buffer at 37℃. The nuclei were labeled with DAPI and observed under a confocal fluorescence microscope.

PI staining and Lactate dehydrogenase (LDH) activity assay. The NMCs were washed by PBS for 3 times and stained with 30μg/ mL PI (Solarbio, Beijing, China) at room temperature for 10-30min under dark conditions. After washing, the cells were anti-stained with DAPI and observed under fluorescence microscope. Then, the PI-positive nuclei were counted and the total of PI-positive cells were expressed as a percentage of total DAPI-positive nuclei (represents the total number of the cells). The LDH assay kit (Jiancheng, Nanjing, Jiangsu, China) was used to determine the activity of LDH released from cell culture medium according to the instructions. After the collected cell culture medium were treated according to the instructions, the absorbance was measured at 450nm wavelength by enzyme labeling instrument (BioTek, Synergy Neo2, USA), and the LDH activity was calculated.

Nuclear and cytoplasmic RNA extraction. Nuclear/Cytosol fractionation kit (Biovision, Inc, CA) was used to separate cytoplasmic and nuclear extracts from cardiomyocytes. According to the instructions, the myocardial myocytes were lysed with cytosol extraction buffer A (CEB-A) and CEB-B at low temperature, centrifuged

at 16000g, and the supernatant was used as cytoplasmic extract. After the precipitation was suspended with nuclear extraction buffer (NEB), the supernatant was used as nuclear extract after vortex centrifugation. Then the RNA was extracted using Trizol (Sigma, St. Louis, Missouri, USA).

2'-O-methylation at the 3'end. 2'-O-methylation at the 3' end was detected by RTL-P[3]. Briefly, using the mir Vana microRNA separation kit (Ambion, AM1556) to extract small RNA samples. The small RNA sample was connected to the 3' RNA adapter by T4-RNA ligase (Takara, 2050A, Beijing, China). Using high (50 µM) or low (0.5 µM) concentration of dNTPs, the ligated products were reverse transcribed to cDNA in the presence of RT primers anchored or unanchored with modified nucleotides. Then the product was amplified by PCR with specific primers and detected in agarose gel.

RNA pulldown and RNA-binding protein immunoprecipitation (RIP) assay. Cardiomyocytes transfected with biotinylated HAAPIR and NC (Gene Pharma, Shanghai, China) were collected, and briefly incubated in lysate buffer containing protease inhibitor. The lysate mixture (10% of total volume) was taken as input and the rest of the mixture was incubated with streptavidin-sepharose beads. Then the beads were cleaned and boiled with loading buffer, and immunoblot was performed. RIP assay was performed using the Megna RIP RNA-binding Protein Immunoprecipitation Kit (Millipore) according to the manufacturer's instructions. In

brief, the cells were lysed with RIP lysis buffer (50mM Tris pH 8.0, 150mM NaCl, 10% Glycerol, 1mM EDTA, 50mM NaF, 0.1% NP-40) containing protease and RNAase inhibitors. and the whole lysate (100 μl) incubated overnight with the primary antibodies or IgG (negative control) coated beads at 4°C. The protein-RNA complexes bound with beads were captured with magnetic protein A/G beads and centrifuged at 12000×g for 5 min at 4°C. The supernatant was discarded, RNA-binding protein were washed, eluted and treated with Proteinase K. The purified RNA was extracted with Trizol reagent and qRT-PCR was conducted.

Western Blot. Total proteins are extracted from tissues or cells using RIPA lysate (Solarbio, R0020, Beijing, China) containing protease inhibitor (Meilunbio, MA0001, Dalian, China). The protein samples were mixed with 4×loading buffer (Solarbio, P1016, Beijing, China) in a ratio of 3:1, and then separated by SDS-PAGE gel electrophoresis and transferred to PVDF membranes. After incubating with 5% BSA at room temperature for 1-3h, the primary antibodies were incubated overnight at 4℃, and the secondary antibody was incubated for 1 h at room temperature. The membranes were scanned and analyzed with Enhanced Chemiluminescence (ECL) detection system (Thermo Scientific, 34080). The following antibodies were used: Tfec (FineTest); Bik (Affinity); ac^4C (Abcam); NAT10 (Zenbio); METTL1 (Affinity); METTL3 (ProteinTech); METTL14 (ProteinTech); WTAP (ProteinTech); DNMT1 (ABclonal); DNMT3A (Abways); DNMT3B (Abways); ALKBH5 (ProteinTech); FTO (ProteinTech); YTHDF1 (Affinity); YTHDF2 (Affinity); TRBP (Affinity).

Quantitative reverse transcription-polymerase chain reaction (qRT-PCR). Total RNA of tissues or cells was extracted with Trizol reagent. Total RNA was reverse transcribed into cDNA, by reverse transcription kit (Takara, RR047A, Beijing, China). The amplification conditions were as follows: 37°C, 15 min; 85°C, 5 s, 4°C. And qRT-PCR was performed using TB Green series (Takara, RR820A, Beijing, China). Amplification conditions: 95°C for 3 min; 95°C 5 s, 60°C 30 s, 40 cycles. The results were normalized to U6 or glyceraldehyde-3-phosphatedehydrogenase (GAPDH) used as internal references. For qRT-PCR analysis, the primer sequences for specific genes are provided in Supplementary Table 4.

ac4C-IP-qPCR. In order to quantify the specific gene level of ac^4C-modification, total RNA was extracted from cardiomyocytes or mouse hearts. Then the total RNA was fragmented, the RNA sample (10% of total volume) was used as the input control and the rest of the RNA sample was incubated with ac^4C-antibody-conjugated beads in immunoprecipitation buffer containing with RNase inhibitor at 4℃ for 2h. The ac^4C-containing RNA sample was eluted from the beads. qRT-PCR was performed with gene-specific primers for both the input control and ac^4C-immunoprecipitated samples.

acRIP-seq. The ac^4C-IP-Seq service was provided by CloudSeq Inc. (Shanghai, China). Briefly, total RNA was extracted from wild type and HAAPIR knockout mice

hearts and then was rRNA depleted with Ribo-zero (Illumina) and then subjected to immunoprecipitation with the GenSeqTM ac4C-IP Kit (GenSeq Inc., China) by following the manufacturer's instructions. RNA was randomly fragmented to ~200 nt by RNA Fragmentation Reagents. Protein A/G beads were coupled to the ac^4C antibody by rotating at room temperature for 1 h. The RNA fragments were incubated with the bead-linked antibodies and rotated at 4°C for 4 h. The RNA/antibody complexes are then digested with Proteinase K and the eluted RNA is purified by phenol:chloroform extraction. RNA libraries for IP and input samples were then constructed with NEBNext® Ultra II Directional RNA Library Prep Kit (New England Biolabs, Inc., USA) by following the manufacturer's instructions. Libraries were qualified using Agilent 2100 bioanalyzer and then sequenced in a Novaseq 6000 platform (Illumina). Paired-end reads were then harvested, and were quality controlled by Q30. After 3' adaptor-trimming and low quality reads removing by cutadapt software (v1.9.3), clean reads of all libraries were aligned to the reference genome by Hisat2 software (v2.0.4). Acetylated sites on RNAs (peaks) were identified by MACS software. Differentially acetylated sites were identified by diffReps. These peaks identified by both softwares overlapping with exons of mRNA were figured out and chosen by home-made scripts.

mRNA-seq. MRNA-Seq high throughput sequencing and subsequent bioinformatics analysis were done by Cloud-Seq Biotech (Shanghai, China). The total RNA was extracted from wild type and HAAPIR knockout mice hearts. Briefly, total RNA (1 μg)

was used for removing the rRNAs using Ribo-Zero rRNA Removal Kits (Illumina, San Diego, CA, USA). RNA libraries were constructed by using rRNA-depleted RNAs with TruSeq Stranded Total RNA Library Prep Kit (Illumina, San Diego, CA, USA) according to the manufacturer's instructions. Libraries were controlled for quality and quantified using the BioAnalyzer 2100 system (Agilent Technologies, Inc., USA). 10 pM libraries were denatured as single-stranded DNA molecules, captured on Illumina flow cells, amplified in situ as clusters and finally sequenced for 150 cycles on Illumina HiSeq Sequencer according to the manufacturer's instructions. Paired-end reads were harvested from Illumina HiSeq 4000 sequencer, and were quality controlled by Q30. After 3' adaptor-trimming and low quality reads removing by cutadapt software (v1.9.3), the high quality clean reads were aligned to the reference genome (UCSC MM10) with hisat2 software (v2.0.4). Then, guided by the Ensembl gtf gene annotation file, cuffdiff software (part of cufflinks) was used to get the gene level FPKM as the expression profiles of mRNA, and fold change and p-value were calculated based on FPKM, differentially expressed mRNA were identified.

Chromatin Immunoprecipitation (ChIP). ChIP analysis were carried out using a chromatin immunoprecipitation assay kit (Millipore, Billerica, MA) and the experiment performed in accordance with the manufacturers protocol as we previously described[8]. In brief, the primary cardiomyocytes were crosslinked with 1% formaldehyde (for 10 min) at room temperature and quenched with ice-cold 0.125M

glycine for 5 min. Then the cells were centrifuged at 2500g for 2 min, washed with PBS, and resuspended in ice-cold cell lysis buffer (kept at 4°C for 1 h). The cell lysate was sonicated to generate chromatin fragments with an average length about 500–800 bp. The samples were precleared using protein A-agarose (Roche) for 1 h at 4°C followed by an overnight incubation with anti-Tfec or anti-mouse IgG at 4°C. The immune complexes were precipitated with protein A-agarose (pre-blocked with 2 μg/ml of salmon sperm DNA at 4 °C overnight) for 4 h. The precipitated DNA fragments were purified using a QIAquick Spin Kit (Qiagen) for PCR assay.

ChIP-seq. CHIP-Seq was provided by CloudSeq Biotech (Shanghai, China). The yield of ChIPed DNA was determined via Quant IT fluorescence assay (Life Technologies) and enrichment efficiencies of ChIP reactions were evaluated by qPCR. Illumina sequencing libraries were generated with NEBNext® Ultra™ DNA Library Prep Kit (New England Biolabs) by following the manufacturer's manual. The library quality was determined by using Agilent 2100 Bioanalyzer (Agilent), and then, subjected to high-throughput 150 base paired-end sequencing on Illumina Novaseq sequencer according to the manufacturer's recommended protocol. Raw data were generated after sequencing, image analysis, base calling and quality filtering on Illumina NovaSeq 6000 sequencer. Firstly, Q30 was used to perform quality control. After adaptor-trimming and low quality reads removing by cutadapt (v1.9.1) software, high quality clean reads were generated. Then these clean reads were aligned to mouse reference genome (MM10) using bowtie2 software (v2.2.4) with default

parameters. Peak calling was performed with MACS software (v1.4.3). Differentially enriched regions were identified by diffReps software (v1.55.4). The enriched peaks were then annotated with the latest UCSC RefSeq database to connect the peak information with the gene annotation.

Statistical Analysis. Data were expressed as the mean ± SEM. Statistical analysis was performed using GraphPad Prism 7.0 (GraphPad Software Inc., San Diego, CA). Student's t-test was used for the comparison of statistical significance between two groups and all tests were determined by unpaired two-sided tests. Comparisons between multiple groups were assessed by one-way ANOVA with Tukey's multiple comparisons test or two-way ANOVA with Bonferroni's multiple comparisons test. The experiments were repeated independently at least three times with similar results and $p < 0.05$ was considered statistically significant.

REFERENCES

1. Wang K, Liu CY, Zhou LY, Wang JX, Wang M, Zhao B, Zhao WK, Xu SJ, Fan LH, Zhang XJ, Feng C, Wang CQ, Zhao YF, Li PF. Apf lncrna regulates autophagy and myocardial infarction by targeting mir-188-3p. *Nature communications*. 2015;6:6779
2. Wang K, Long B, Zhou L, Liu F, Zhou Q, Liu C, Fan Y, Li P. Carl lncrna inhibits anoxia-induced mitochondrial fission and apoptosis in cardiomyocytes by impairing

mir-539-dependent phb2 downregulation. *Nature communications*. 2014;5:3596

3. Gao XQ, Zhang YH, Liu F, Ponnusamy M, Zhao XM, Zhou LY, Zhai M, Liu CY, Li XM, Wang M, Shan C, Shan PP, Wang Y, Dong YH, Qian LL, Yu T, Ju J, Wang T, Wang K, Chen XZ, Wang YH, Zhang J, Li PF, Wang K. The pirna chapir regulates cardiac hypertrophy by controlling mettl3-dependent n(6)-methyladenosine methylation of parp10 mrna. *Nature cell biology*. 2020;22:1319-1331

4. Zhou LY, Zhai M, Huang Y, Xu S, An T, Wang YH, Zhang RC, Liu CY, Dong YH, Wang M, Qian LL, Ponnusamy M, Zhang YH, Zhang J, Wang K. The circular rna acr attenuates myocardial ischemia/reperfusion injury by suppressing autophagy via modulation of the pink1/ fam65b pathway. *Cell Death Differ*. 2019;26:1299-1315

5. Shevchenko A, Tomas H, Havlis J, Olsen JV, Mann M. In-gel digestion for mass spectrometric characterization of proteins and proteomes. *Nature protocols*. 2006;1:2856-2860

6. Straub V, Rafael JA, Chamberlain JS, Campbell KP. Animal models for muscular dystrophy show different patterns of sarcolemmal disruption. *The Journal of cell biology*. 1997;139:375–385

7. Lee GJ, Yan L, Vatner DE, Vatner SF. Mst1 inhibition rescues β_1-adrenergic cardiomyopathy by reducing myocyte necrosis and non-myocyte apoptosis rather than myocyte apoptosis. *Basic research in cardiology*. 2015;110:7

8. Wang K, An T, Zhou LY, Liu CY, Zhang XJ, Feng C, Li PF. E2f1-regulated mir-30b suppresses cyclophilin d and protects heart from ischemia/reperfusion injury and necrotic cell death. *Cell Death Differ*. 2015;22:743-754

9. Wang K, Long B, Jiao JQ, Wang JX, Liu JP, Li Q, Li PF. Mir-484 regulates mitochondrial network through targeting fis1. *Nature communications*. 2012;3:781

SUPPLEMENTAL FIGURE LEGENDS

Supplementary Figure 1. Identification of HAAPIR in cardiomyocytes. (a and b) The expression levels of highly upregulated piRNAs (selected from previous piRNA microarray data) in I/R injured mice hearts determined by qPCR (n=3 independent experiments). **(c)** qPCR result showing the expression level of DQ542443 in different cell types isolated from the mouse heart (myocytes, fibroblasts) (n=6 independent experiments). **(d, e)** Isolated neonatal mice cardiomyocytes were transfected with HAAPIR agomir (HAAPIR) or its negative control (NC) for 24 h. **(d)** Apoptosis was determined by the terminal deoxynucleotidyl transferase dUTP nick end labeling (TUNEL) assay. DAPI indicates Nucleus. Bar=50µm. **(e)** Cardiomyocytes were staining with MitoTracker Red (red) /DAPI (blue). Bar=10µm. Data are presented as Mean ± SEM. Two-sided Student's t-test (a-c).

Supplementary Figure 2. Knockdown of HAAPIR attenuates hypoxia-reoxygenation (H/R)-induced cardiomyocyte apoptosis. (a) Isolated neonatal mice cardiomyocytes were subjected to H/R at indicated time. QPCR analysis showing expression level of HAAPIR at indicated time points (n=6 independent experiments). **(b-f)** Isolated neonatal mice cardiomyocytes were transfected with HAAPIR antagomir (anta) or its negative control (anta-NC) for 24 h and then cells were treated with H/R. **(b)** qPCR analysis of the expression level of

HAAPIR (n=6 independent experiments). **(c)** Apoptosis was determined by the terminal deoxynucleotidyl transferase dUTP nick end labeling (TUNEL) assay. DAPI indicates Nucleus. Bar=50μm. **(d)** Quantitative analysis of the percentage of apoptotic cells (n=6 independent experiments). **(e)** The percentage of PI positive cells calculated from cardiomyocytes transfected with anta or anta-NC. (n=5 independent experiments). Bar=50μm. **(f)** The lactate dehydrogenase (LDH) activity in anta or anta-NC transfected cardiomyocytes (n=3 independent experiments). Data are presented as Mean ± SEM. All data were analyzed using one-way ANOVA.

Supplementary Figure 3. HAAPIR binds to NAT10. (a and **b)** RNA immunoprecipitation (RIP) was performed in isolated cardiomyocytes using antibodies against METTL1, METTL3, METTL14, NAT10, WTAP, DNMT1, DNMT3A, DNMT3B, ALKBH1, ALKBH5, FTO, YTHDF1, YTHDF2 and TRBP. The binding of HAAPIR to NAT10 was confirmed by qPCR (n=5 independent experiments). **(c)** Quantitative real-time PCR (qPCR) analysis showing the mRNA level of NAT10 in isolated cardiomyocytes treated with or without HAAPIR agomir or agomir-NC (NC) (n=5 independent experiments). **(d)** Representative western blot showing expression of NAT10 in isolated cardiomyocytes treated with or without HAAPIR agomir or NC. **(e)** qPCR analysis showing the mRNA level of NAT10 in HAAPIR knockout (KO) and wild-type (WT) mice hearts (n=7 mice per group). **(f)** Representative western blot showing expression of NAT10 in HAAPIR KO and WT mice hearts. Data are presented as Mean ± SEM. One-way ANOVA test (a-c) or

two-sided Student's t-test (e).

Supplementary Figure 4. Epitranscriptome and transcriptome analyses in HAAPIR knockout (KO) mice hearts. (a-c) AcRIP-seq was performed in HAAPIR KO and wild-type (WT) mice hearts. **(a)** Pie chart depicting the distribution of ac^4C peaks in different transcript segments in HAAPIR KO and WT mice hearts. **(b** and **c)** Gene ontology (GO) analysis of enriched terms in biological processes associated with HAAPIR target genes. **(d)** Representative western blot (upper panel) and quantitative real-time PCR (qPCR) analysis (lower panel, n=6 independent experiments) showing the expression levels of Tfec in HAAPIR KO and WT mice hearts. Data are presented as Mean ± SEM. Two-sided Student's t-test (d).

Supplementary Figure 5. Inhibition of Tfec attenuates cardiomyocyte apoptosis in vitro and in vivo. (a) Isolated neonatal mice cardiomyocytes were transfected with Tfec siRNA (si-Tfec) or its negative control (si-NC) for 24 h and then cells were treated with H_2O_2 for an additional 24 h. Apoptosis was determined by the terminal deoxynucleotidyl transferase dUTP nick end labeling (TUNEL) assay. DAPI indicates Nucleus. Bar=50μm. **(b)** AAV9-Tfec-shRNA (shTfec) or AAV9-scrambled control (shCTRL) were injected into mice and I/R induced heart injury was performed. Apoptosis was determined by the Terminal deoxynucleotidyl transferase (TdT) dUTP Nick-End Labeling (TUNEL) assay. DAPI indicates Nucleus. Immunostaining of cTnT labels cardiomyocytes. Bar=25μm.

Supplementary Figure 6. Inhibition of Tfec attenuates H/R-induced cardiomyocyte apoptosis. (a-d) Isolated neonatal mice cardiomyocytes were transfected with Tfec siRNA (si-Tfec) or its negative control (si-NC) for 24 h and then cells were treated with H/R. **(a)** Representative western blot showing the expression of Tfec. **(b)** The percentage of apoptotic cardiomyocytes was determined by TUNEL assay (n=6 independent experiments). Bar=50µm. **(c)** The percentage of PI positive cells calculated from cardiomyocytes transfected with si-Tfec or si-NC (n=5 independent experiments). Bar=50µm. **(d)** The lactate dehydrogenase (LDH) activity in si-Tfec or si-NC transfected cardiomyocytes (n=3 independent experiments). Data are presented as Mean ± SEM. One-way ANOVA test (b-d).

Supplementary Figure 7. HAAPIR promotes Tfec expression and cardiomyocyte apoptosis by NAT10. (a and **b)** Isolated neonatal cardiomyocytes were transfected with HAAPIR antagomir (anta) or its negative control (anta-NC) for 24 h and then cells were treated with H_2O_2 for an additional 24 h. The mRNA **(a)** and protein **(b)** levels of Tfec were detected by quantitative real-time PCR (qPCR) and western blot assay, respectively. (n=5 independent experiments). **(c** and **d)** NAT10 siRNA (siNAT10) or its control (siNC) and HAAPIR agomir (HAAPIR) were transfected into cardiomyocytes. The protein levels of Tfec **(c)** and the percentage of apoptotic cardiomyocytes **(d)** were detected, respectively. (n=5 independent experiments). Data are presented as Mean ± SEM. One-way ANOVA test (a, d).

Supplementary Figure 8. Tfec regulates Bik expression during cardiomyocyte apoptosis. (a) Scatter plot of differential expression of mRNAs assessed from RNA-seq data in cardiomyocytes transfected with flag-tagged Tfec and treated with or without H_2O_2. Red dots denote up-regulated genes and green dots denote down-regulated genes. **(b)** Combining the results of differentially Tfec-enriched gene regions identified in the ChIP-seq dataset (left circle) and differentially expressed mRNAs in RNA-Seq analysis (right circle). 376 genes were present in both datasets. **(c** and **d)** Isolated neonatal cardiomyocytes were transfected with Bik siRNA (siBik) or its negative control (siNC) for 24 h and then cells were treated with H_2O_2 for an additional 24 h. Quantitative analysis of the percentage of apoptotic cells **(c)** and the percentage of cells with fragmented mitochondria **(d)** were determined, respectively (n=5 independent experiments). **(e)** NAT10 siRNA (siNAT10) or its control (siNC) and HAAPIR agomir (HAAPIR) were transfected into cardiomyocytes. The protein levels of Bik were detected (n=5 independent experiments). Data are presented as Mean ± SEM. One-way ANOVA test (c, d).

Supplementary Figure 9. Tfec and Bik function as the downstream molecules of HAAPIR during cardiomyocyte apoptosis and myocardial injury. (a-d) AAV9-Tfec-shRNA (shTfec) or AAV9-scrambled control (shCTRL) were injected into 8-weeks old HAAPIR KO mice and I/R injury was performed. **(a)** The protein levels of Bik were detected by western blot assay. **(b)** The percentage of apoptotic

cardiomyocytes was determined by TUNEL assay (n=6 mice per group). **(c)** The infarct sizes after I/R induced heart injury were indicated by the ratio of infarct area (INF)/ left ventricle (LV) (n=6 mice per group). Bar=2mm. **(d)** Heart function measured by left ventricle fractional shortening (FS) using echocardiography (n=6-7 mice per group). Data are presented as Mean ± SEM. One-way ANOVA test (b-d).

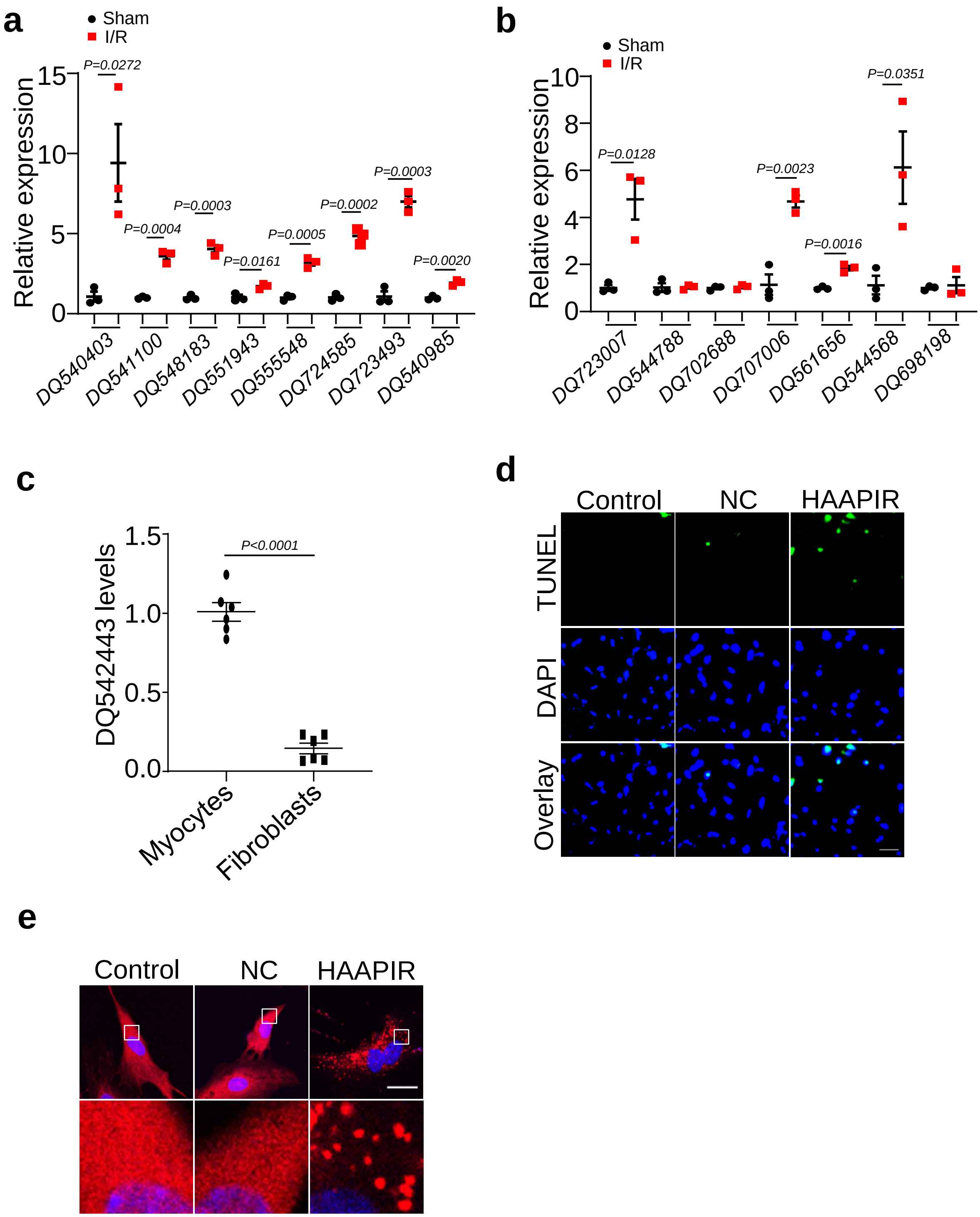
a
Sham
I/R
Relative expression
15
10
5
0
P=0.0272
P=0.0004
P=0.0003
P=0.0161
P=0.0005
P=0.0002
P=0.0003
P=0.0020
DQ540403
DQ541100
DQ548183
DQ551943
DQ555548
DQ724585
DQ723493
DQ540985
b
Sham
I/R
Relative expression
10
8
6
4
2
0
P=0.0128
P=0.0023
P=0.0016
P=0.0351
DQ723007
DQ544788
DQ702688
DQ707006
DQ561656
DQ544568
DQ698198
c
DQ542443 levels
1.5
1.0
0.5
0.0
P<0.0001
Myocytes
Fibroblasts
d
Control
NC
HAAPIR
TUNEL
DAPI
Overlay
e
Control
NC
HAAPIR

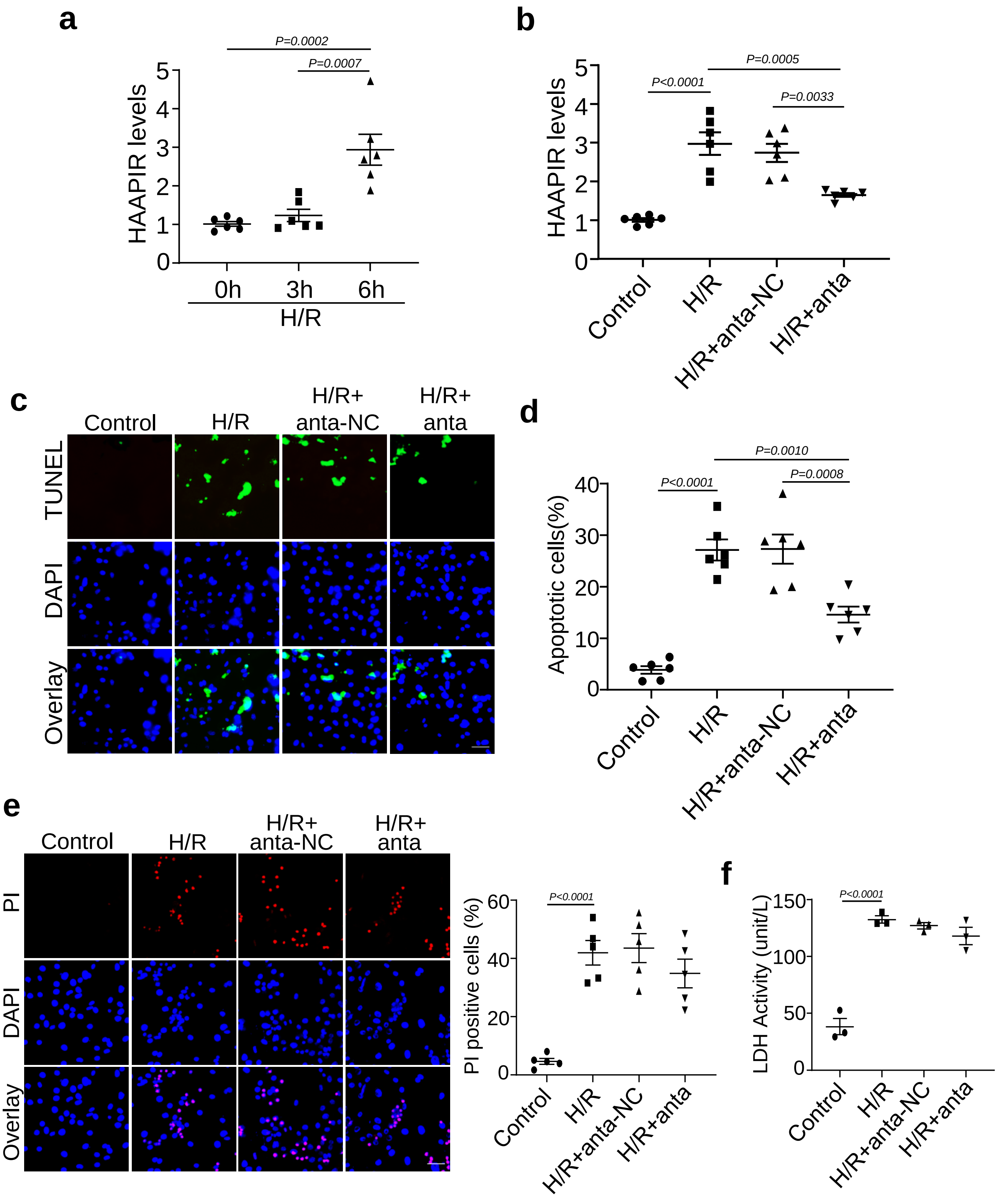
a
P=0.0002
P=0.0007
HAAPIR levels
0h
3h
6h
H/R
b
HAAPIR levels
P<0.0001
P=0.0005
P=0.0033
Control
H/R
H/R+anta-NC
H/R+anta
c
Control
H/R
H/R+ anta-NC
H/R+ anta
TUNEL
DAPI
Overlay
d
Apoptotic cells(%)
P<0.0001
P=0.0010
P=0.0008
Control
H/R
H/R+anta-NC
H/R+anta
e
Control
H/R
H/R+ anta-NC
H/R+ anta
PI
DAPI
Overlay
PI positive cells (%)
P<0.0001
Control
H/R
H/R+anta-NC
H/R+anta
f
LDH Activity (unit/L)
P<0.0001
Control
H/R
H/R+anta-NC
H/R+anta

Supplementary Fig. 3

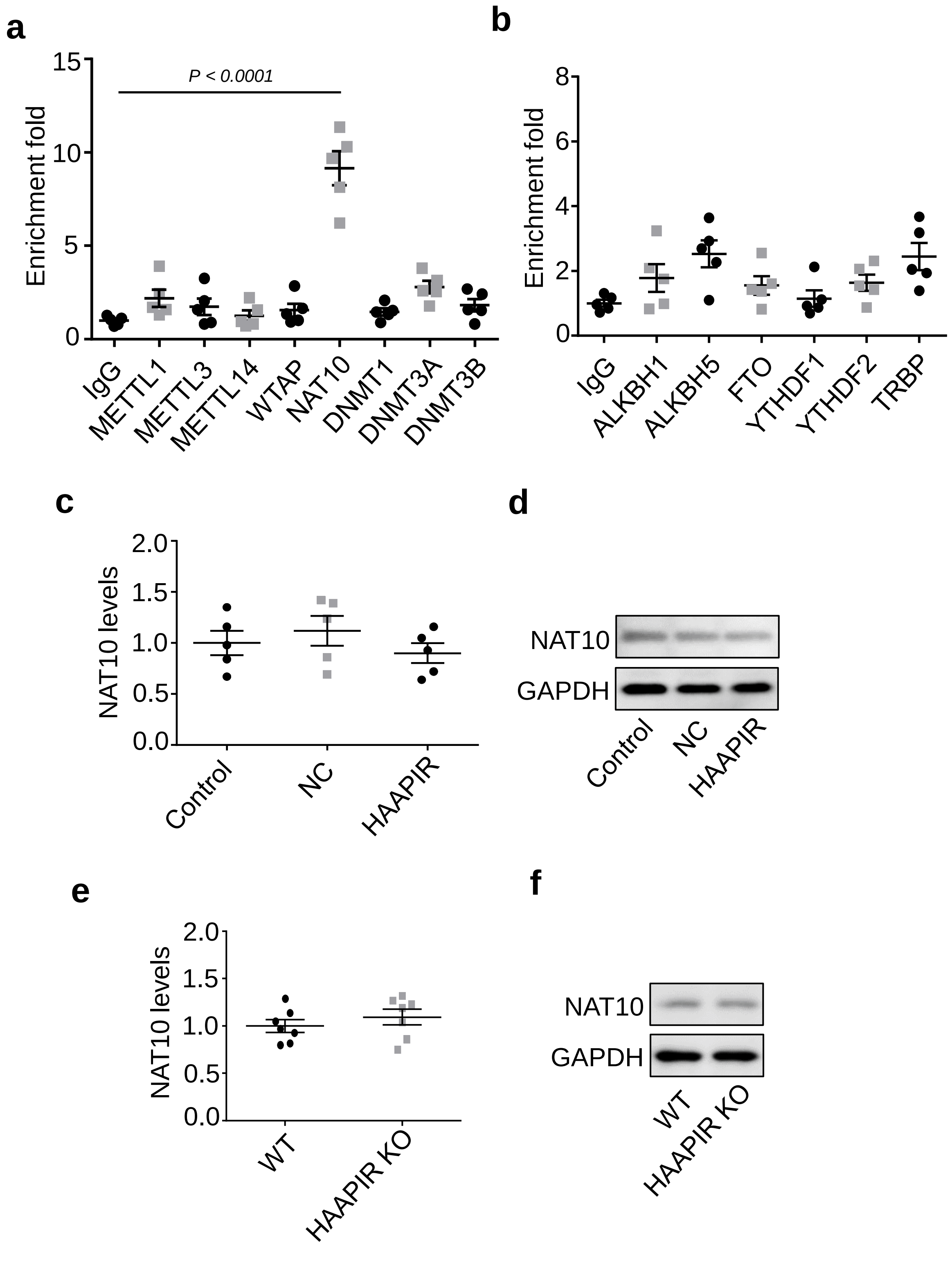

Supplementary Fig. 4

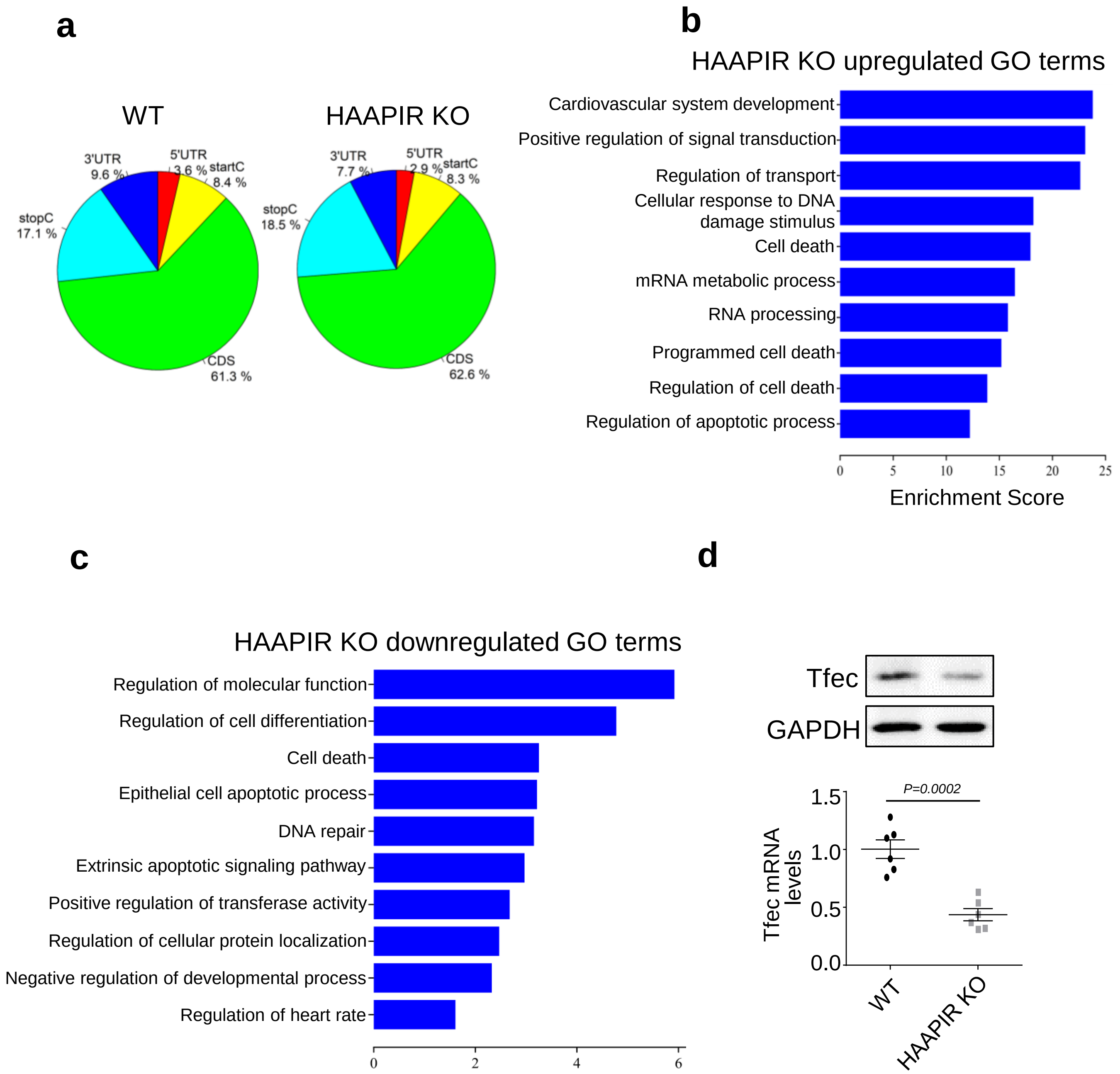

a

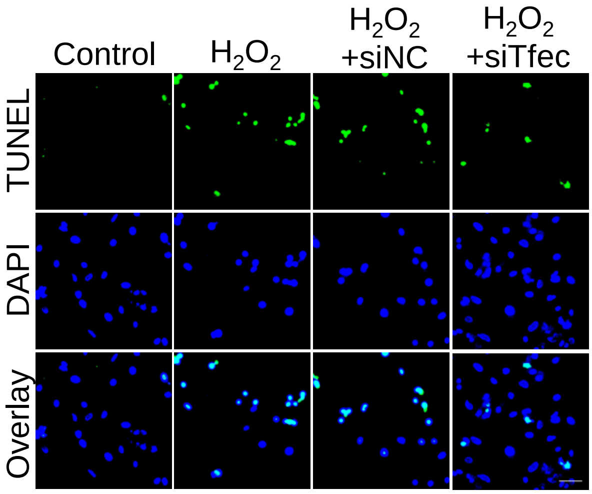
Control
H_2O_2
H_2O_2 +siNC
H_2O_2 +siTfec
TUNEL
DAPI
Overlay

b

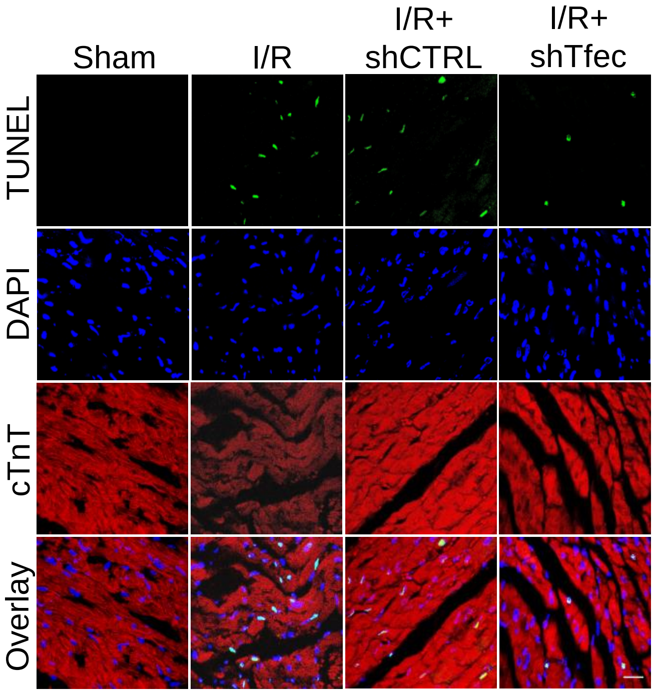
Sham
I/R
I/R+ shCTRL
I/R+ shTfec
TUNEL
DAPI
cTnT
Overlay

Supplementary Fig. 6

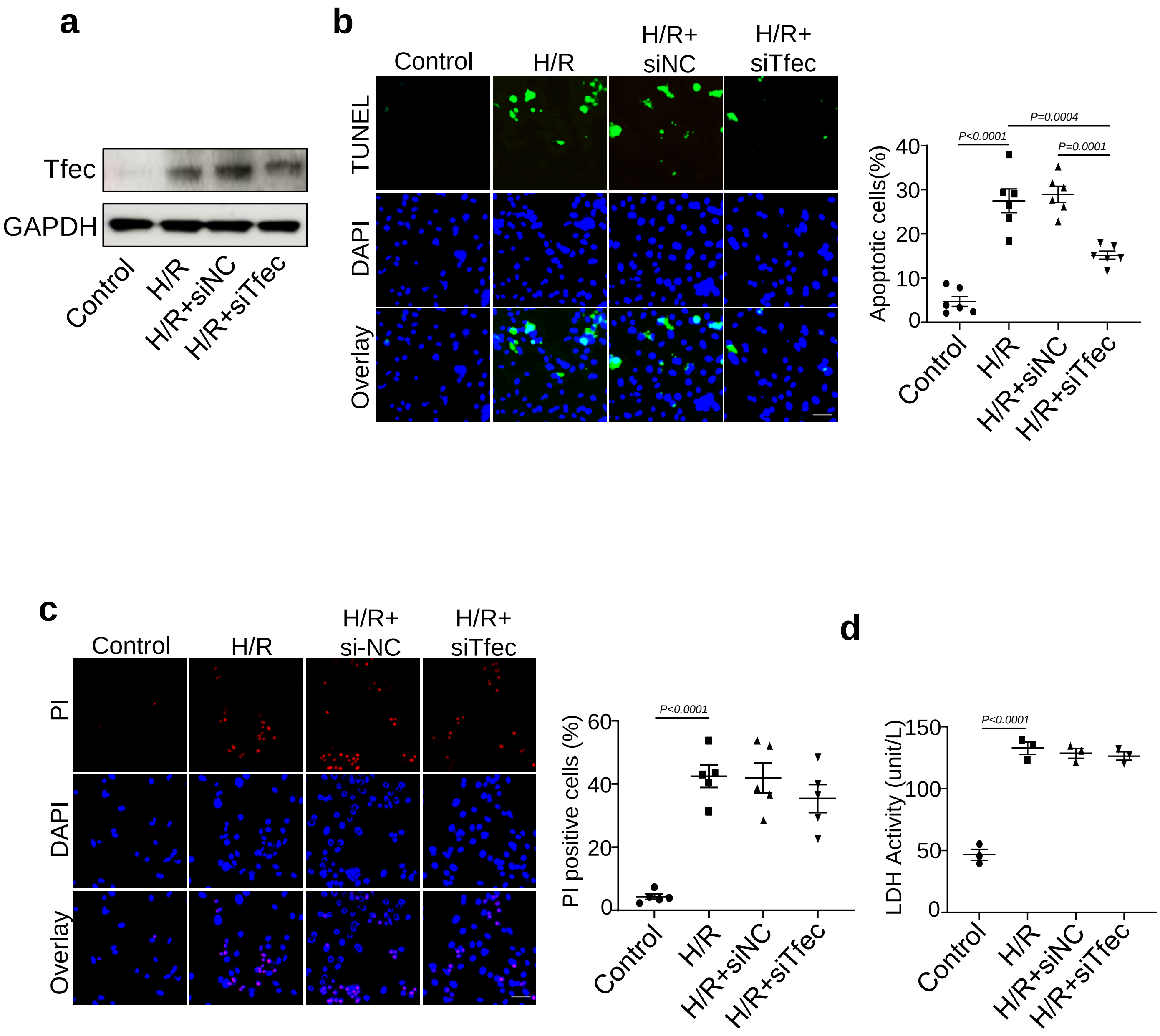

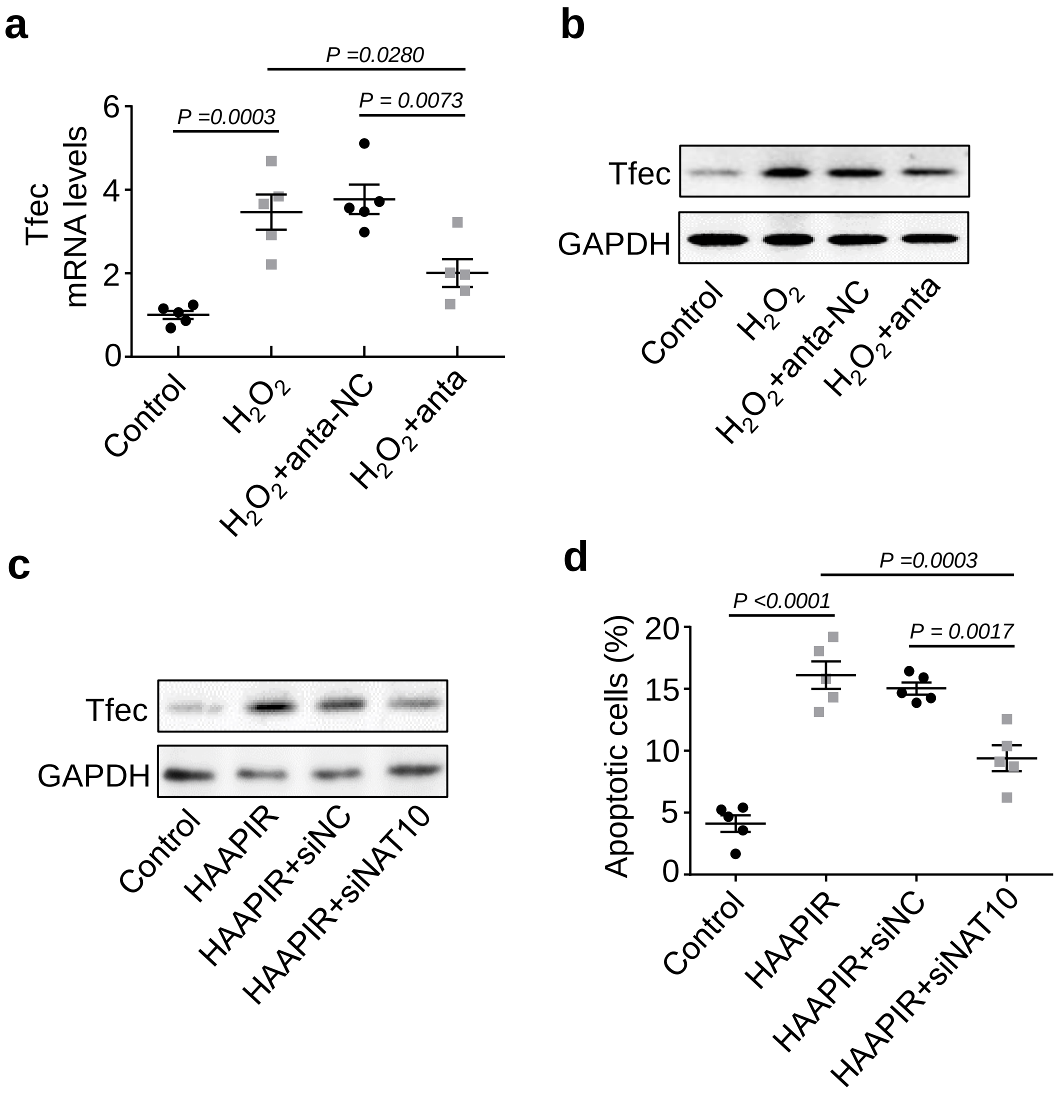
a
P =0.0280
P = 0.0073
P =0.0003
Tfec mRNA levels
6
4
2
0
Control
H2O2
H2O2+anta-NC
H2O2+anta
b
Tfec
GAPDH
Control
H2O2
H2O2+anta-NC
H2O2+anta
c
Tfec
GAPDH
Control
HAAPIR
HAAPIR+siNC
HAAPIR+siNAT10
d
P =0.0003
P <0.0001
P = 0.0017
Apoptotic cells (%)
20
15
10
5
0
Control
HAAPIR
HAAPIR+siNC
HAAPIR+siNAT10

Supplementary Fig. 8

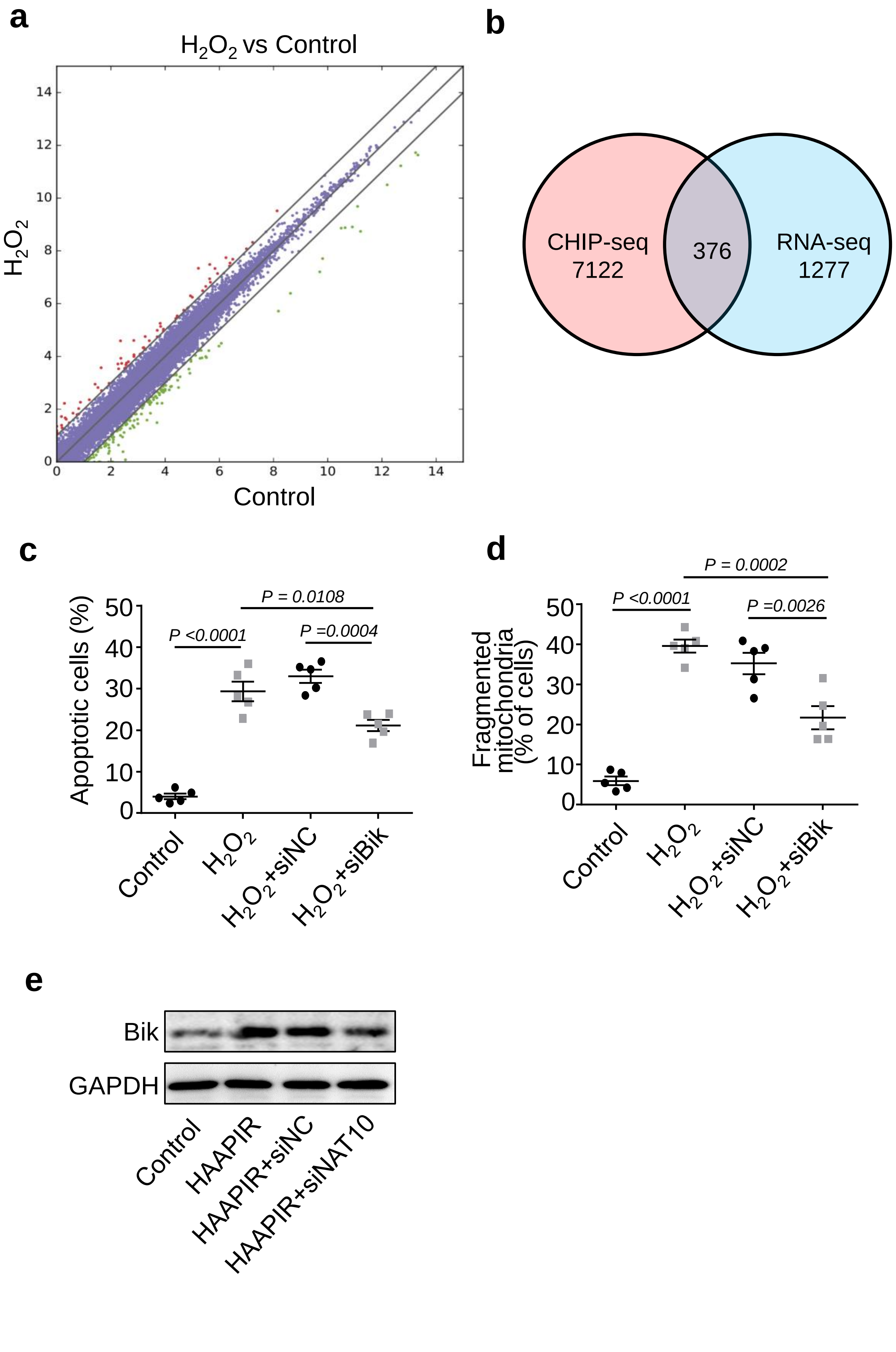

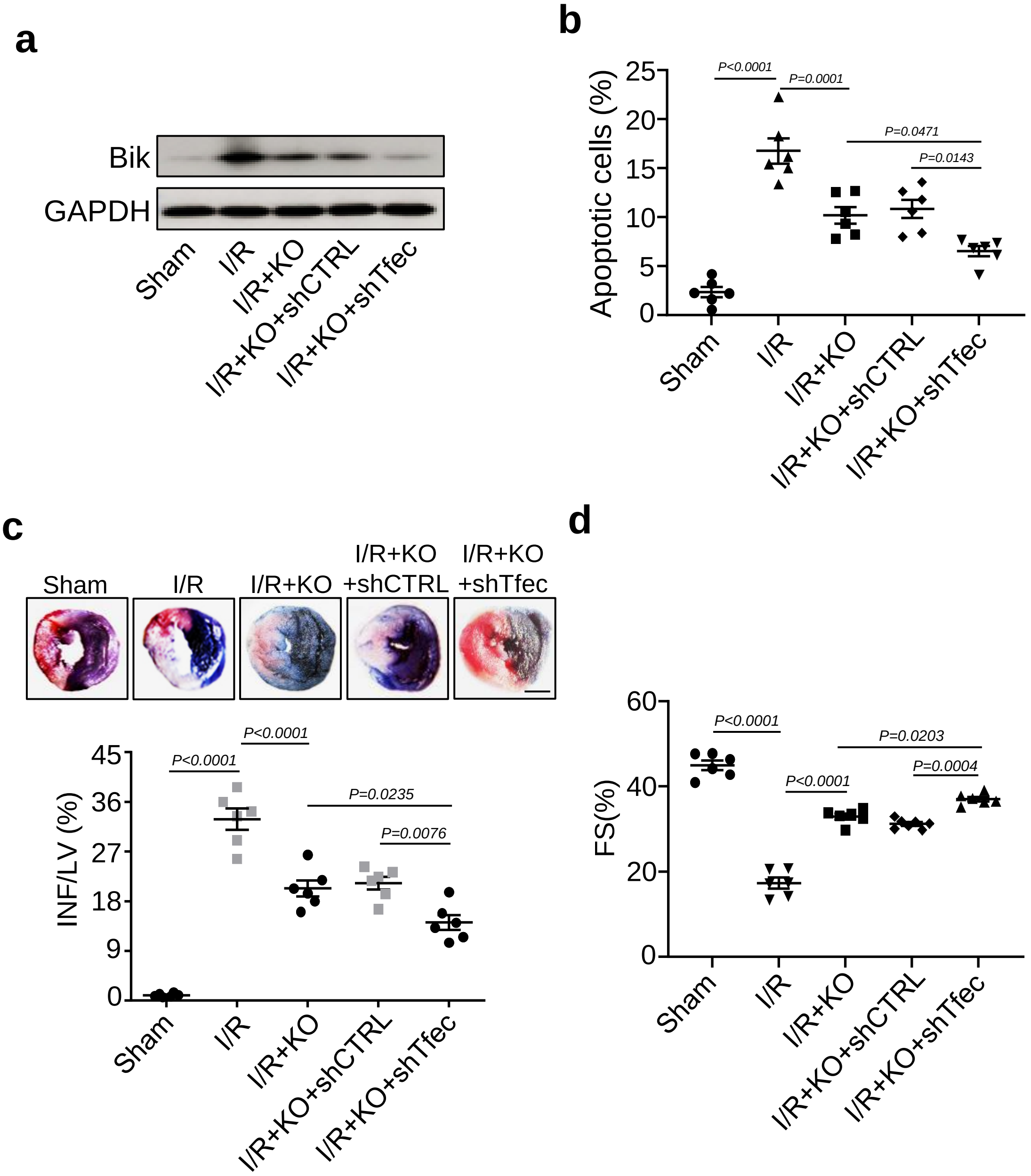
a
Bik
GAPDH
Sham
I/R
I/R+KO
I/R+KO+shCTRL
I/R+KO+shTfec
b
Apoptotic cells (%)
P<0.0001
P=0.0001
P=0.0471
P=0.0143
c
Sham
I/R
I/R+KO
I/R+KO +shCTRL
I/R+KO +shTfec
INF/LV (%)
P<0.0001
P<0.0001
P=0.0235
P=0.0076
d
FS(%)
P<0.0001
P=0.0203
P=0.0004
P<0.0001
